# Psychological responses to acute exercise in patients with stress-induced exhaustion disorder: a cross-over randomized trial

**DOI:** 10.1186/s12888-025-06484-1

**Published:** 2025-01-24

**Authors:** Jenny Kling, Robert Persson Asplund, Örjan Ekblom, Victoria Blom

**Affiliations:** 1 Department of Physical Activity and Health, The Swedish School of Sport and Health Sciences, Lidingövägen 1, 114 33 Stockholm, Sweden; 2 Department of Behavioural Sciences and Learning, Linköping University, Linköping, Sweden; 3 Department of Neurobiology, Care Sciences and Society, Division of Nursing, Research Group: Health Promotion Among Children and Youth, Karolinska Institute, Stockholm, Sweden; 4 Division of Insurance Medicine, Department of Clinical Neuroscience, Karolinska Institute, Stockholm, Sweden

**Keywords:** Exercise, Acute, Exhaustion disorder, Stress, Fatigue, Energy, Anxiety, Exercise intensity

## Abstract

**Background:**

Understanding psychological responses to acute exercise, defined as a single bout of physical exercise, in clinical populations is essential for developing tailored interventions that account for the psychological benefits and challenges of exercise. Given its effectiveness in reducing symptoms in various psychological disorders, exercise should be further explored in Exhaustion Disorder ICD-10-SE: F43.8A (ED), characterized by persistent exhaustion following long-term psychosocial stress. Currently, no studies address the psychological responses to acute exercise in ED patients.

**Aims:**

This study aims to (1) compare the psychological responses to acute exercise between ED patients and healthy controls and (2) assess response differences between low and moderate exercise intensities.

**Methods:**

We conducted a two-armed cross-over trial comparing ED patients (n = 30) and healthy controls (n = 30). Participants completed a 22-min exercise at low or moderate intensity on a cycle ergometer, on separate occasions, in randomized order. The primary outcome was perceived fatigue (POMS); secondary outcomes included feelings of energy, anxiety, stress, exertion, and psychological discomfort, measured before, during, and up to 24 h post-exercise. Exercise effects were assessed using repeated measures analysis of variance.

**Results:**

ED patients reported higher levels of exertion, psychological discomfort, fatigue, anxiety, and stress but lower energy throughout the trial compared to controls. Unlike controls, the ED group showed significant fatigue and stress reductions post-exercise (*p* < 0.05). Additionally, ED patients showed a more elevated energy after moderate-intensity exercise compared to controls (*p* < 0.05). Both groups experienced anxiety reductions post-exercise, with no group interactions over time. No differences were observed between pre- and 6 or 24 h post-exercise in any variables. The only intensity effect (*p* < 0.05) in the ED patients was a more pronounced energy decline 30 min after moderate-intensity exercise.

**Conclusions:**

A 22-min exercise session was perceived as more strenuous by patients with exhaustion disorder (ED) and generated greater improvements in feelings of fatigue, energy, and stress compared to healthy individuals without delayed negative effects. These findings highlight the specific psychological responses in ED to exercise and can inform intervention design tailored specifically to this population.

**Trial registration:**

The study was retrospectively registered on 05/30/2024 at Clinical Trials.gov, with trial registration number 2022–04943-01.

**Supplementary Information:**

The online version contains supplementary material available at 10.1186/s12888-025-06484-1.

## Background

Consequences of chronic psychosocial stress have increasingly contributed to the global issue of mental ill health. Exhaustion disorder (ED) is the primary reason for long-term sick leave in Sweden for all psychiatric and somatic diseases [1]. ED has been recognized in the Swedish edition of the International Statistical Classification of Diseases since 2005 (SE-ICD-10, code F43.8A.) [2]. The diagnosis is characterized by persistent mental and physical exhaustion and noticeably reduced energy, resulting from identifiable psychosocial stressors that have been present for at least six months without sufficient recovery [3]. Fatigue is a core clinical characteristic of ED, and it has recently been suggested that ED symptoms might be better understood as transdiagnostic symptoms of fatigue rather than a diagnosis-specific pathology [4].

The concept of central fatigue, as described by Leavitt and DeLuca [5], highlights the complexity of fatigue as a multifaceted construct that includes cognitive components such as the difficulty in initiating and sustaining attentional tasks and physical activities requiring self-motivation. This aligns with the understanding of ED where fatigue is not just a symptom but a central feature that impacts cognitive and physical functioning.

ED shares several symptomatic features with burnout but differs by including preceding non-work-related stressors. Consequently, individuals with ED also score high on burnout [3]. Given that economic and societal costs associated with stress-related disorders, primarily from sick leave and productivity loss, have been estimated at approximately 187 billion USD in the Western World [6], it is imperative that research focuses on feasible and effective treatments [7].

A recent umbrella review showed that regular exercise is effective in symptom reduction of depression, anxiety, and distress both across multiple populations and specifically for patients with depression and different anxiety- and stress disorders [8]. As ED shares symptoms with several of these conditions, it may be assumed that exercise may hold beneficial effects also for ED. However, the benefits of regular exercise for burnout [9] and ED [7] remain inconclusive. Studies conducted on ED patients and exercise interventions have shown that adding exercise to a regular treatment gave small [10] or no effects on symptom severity [11].

An important consideration in developing feasible and effective exercise-based interventions for ED is the psychological effects of acute exercise, defined as a single bout of exercise, given the likely central role of immediate exercise effects on affect and emotion in motivating sustained exercise behavior [12]. Previous research has shown that a 30-min aerobic exercise bout can reduce mental health symptoms post-exercise across several psychiatric conditions. In patients with depression, it significantly decreased depression symptoms, regardless of exercise intensity [13]. Similarly, for patients with generalized anxiety disorder (GAD), running at a vigorous intensity significantly reduced anxiety and elevated energy compared to quiet rest [14]. Additionally, moderate-intensity exercise had anxiolytic and anti-panic effects compared to quiet rest in patients with panic disorder [15].

In contrast, a meta-analysis on the effects of acute exercise effects in individuals with myalgic encephalomyelitis/chronic fatigue syndrome (ME/CFS) found increased fatigue post-exercise, with more pronounced elevations several hours afterward [16]. Although ED and ME/CFS share several similarities, such as severe exhaustion, sleep disturbances, and cognitive difficulties, one key difference is the post-exertional malaise in ME/CFS [17]. Loy et al. [18] highlight that acute exercise can increase energy and reduce fatigue but that the effects are influenced by exercise intensity and duration. Their findings suggest that energy and fatigue are separate but related states and that their changes arise through different mechanisms. This supports the idea that exercise could potentially have beneficial effects on fatigue in ED. However, further research is needed to confirm these effects specifically in ED.

To our knowledge, no prior study has examined the psychological responses to acute exercise in individuals with ED. Consequently, the present study aimed to compare the psychological responses to acute exercise between patients with ED and a healthy control group. A second objective was to compare these responses across two exercise intensities: low and moderate. By comparing the acute exercise responses of healthy individuals and those with ED, we aimed to increase the understanding of the specific psychological benefits and challenges exercise may present at different intensities for individuals with a chronic condition like ED. This design facilitates a comparison of psychological responses, providing insights into typical exercise reactions in the ED group compared to those of non-clinical individuals.

The focus was to assess the exercise-related effects on the primary outcome fatigue, as well as the secondary outcomes energy, anxiety, stress, psychological discomfort, and perceived exertion, as these are central aspects of ED. Increased knowledge may support the development of feasible and effective treatment strategies for this burdened patient group.

## Methods

### Study design and participants

**Fig. 1:**
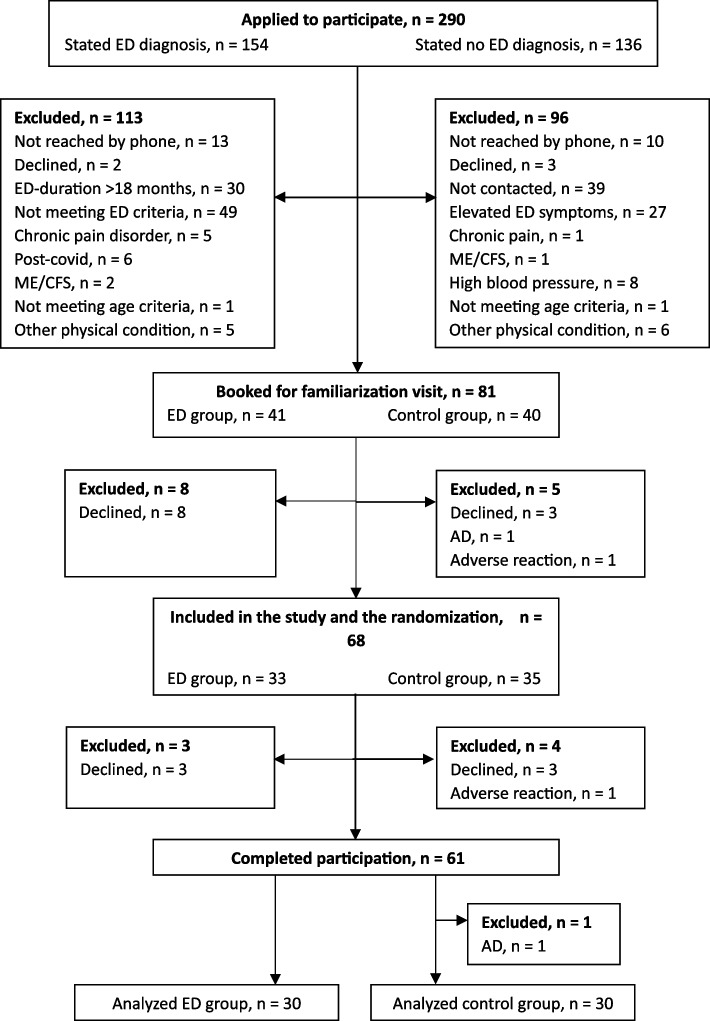
Flowchart showing the inclusion/exclusion process. ED, exhaustion disorder; ME/CFS, myalgic encephalomyelitis/chronic fatigue syndrome; AD, adjustment disorder. Adverse reactions: one control participant experienced chest pain, and another groin pain, upon which the exercise was immediately terminated, further participation ceased, and the participants were followed up with appropriate action

To examine the psychological responses to acute exercise in individuals with ED compared to healthy individuals, we conducted a two-armed cross-over trial comparing low and moderate exercise intensities in two groups: individuals diagnosed with ED and a healthy control group. Based on power calculations for the primary outcome (fatigue), a sample size of 30 patients with ED and 30 control participants would provide a power of 95% with a two-sided 0.05 significance level and a medium effect size. Participants were recruited through advertisements via Facebook, Instagram, and LinkedIn. Interested individuals followed an online link to complete a screening questionnaire that contained measures of ED and burnout, along with questions on mental and general health. Karolinska Exhaustion Disorder Scale (KEDS) was developed for the assessment of ED symptoms [19], and the proposed cut-off of 19 for discriminating between healthy subjects and patients with ED was used. Those who appeared eligible were contacted via telephone and were invited to fill out a baseline questionnaire and come to the research lab for a familiarization visit. During this visit, all participants underwent the Mini International Neuropsychiatric Interview 6.0.0. (M.I.N.I.) [20] complemented by questions targeting stress-related disorders in line with ICD-11 criteria [21]. See Fig. 1 for information on participant flow.

Inclusion criteria for all participants were: (1) age 35–55 years, (2) fluent in Swedish, and (3) no medical contraindication for participation in exercise. Exclusion criteria for all participants were: (1) ME/CFS, (2) chronic pain disorder, (3) recent post-covid, (4) current suicide risk, (5) current drug use or addiction, (6) current or previous bipolar disorder, (7) current or previous psychosis disorder, (8) untreated hypothyroidism, (9) blood pressure > 200/110, (10) medication with beta-blockers, or (11) other known somatic comorbidity that may affect physical and mental response. For inclusion in the ED group, inclusion criteria were: (1) a confirmed diagnosis of ED obtained through regular healthcare, (2) current ED symptomology based on the questionnaires and the diagnostic interview, assessed by a licensed psychologist experienced in working with the patient group, (3) an ED diagnosis not older than 18 months, to ensure a more homogeneous group, reducing variability and improving diagnostic accuracy and response reliability, and (4) not in the acute phase of ED, defined as the initial stage of illness, marked by intensified severe fatigue and inability to function, lasting from several days to weeks [22]. The control group was required to have self-reported good health and none of the specific ED criteria.

All participants received written information about the study, were given an opportunity to ask questions, and provided informed consent before inclusion. The study was approved by the Swedish Ethical Review Authority (Approval Nr. 2022–04943-01). Participants were compensated with 1500 SEK upon completion for expenses related to participation, such as travel costs, leave of absence, etc. The study was retrospectively registered on 05/30/2024 at Clinical Trials.gov, with trial registration number 2022–04943-01.

### Baseline measures

Before the familiarization visit, each participant completed an online questionnaire with sociodemographic and general health information, which was used for comparisons between the two groups. The following questionnaires were included: Saltin-Grimby Physical Activity Level Scale (SGPALS) [23], Karolinska Exhaustion Disorder Scale (KEDS) [19], a short version of the Shirom-Melamed Burnout Questionnaire/Measure (SMBM-12) [24], the trait subscale of The State-Trait Anxiety Inventory (STAI-Y2) [25], Patient Health Questionnaire (PHQ-9) [26], and Pittsburgh Sleep Quality Index (PSQI) [27].

### Procedure

The data collection took place from February 2023 to January 2024 in a laboratory setting at The Swedish School of Sport and Health Sciences (GIH), with each participant attending on three separate days, with a minimum of one week between sessions. We provided transportation to GIH by taxi to minimize variations in physical activity before the test. The first visit served both to familiarize participants with the setting and research team and to conduct pre-randomization assessments. This included the structured clinical interview previously described, plus measures of blood pressure and body mass. Blood pressure was measured using Digital Blood Pressure Monitor HEM-907 (OMRON, Kyoto, Japan) in a sitting position after at least 20 min of seated rest. Further, a submaximal cardiorespiratory fitness test was performed on a calibrated mechanically braked cycle ergometer (Model 828E, Monark, Varberg, Sweden) to estimate maximal aerobic capacity (V̇O_2_ max) [28]. Before this fitness test, participants were verbally informed about the procedures and introduced to Borg’s 6–20 rating of perceived exertion (RPE) scale [29] and Tanner’s Subjective Units of Distress scale (SUDS) [30] using visual analog scales (VAS). The fitness test began after individual adjustments of the cycle’s seat and handlebars and continued for approximately 8 min. The cycling work rate for the first level was set to 32 Watts work rate for 4 min, then increased to a personalized higher work rate for approximately 4 min. The personalized higher work rate aimed at corresponding to a perceived exertion of 13–16 (somewhat hard to hard) using RPE. The pedal frequency was maintained at 60 RPM throughout the test. V̇O_2_ max was estimated using heart rate data collected during the test via an H10 heart rate sensor and Vantage M2 monitor (Polar, Kempele, Finland). The estimated V̇O_2_ max was used to determine the intensity level for each participant in the subsequent trial conditions. The familiarization visit lasted approximately 90 min, and each of the two experimental visits lasted for about 120 min.

### Study outcomes

To assess the effects of exercise on subjective transient feelings of fatigue, energy, anxiety, and stress, the following measures were included at the five time points immediately before, immediately after, 30 min after, 6 h after, and 24 h after the exercise bout:

#### Fatigue and energy

Previous research shows that acute exercise affects energy and fatigue [31], which are also core symptom areas in ED (ICD-10-SE). Feelings of energy and fatigue were measured with the vigor and fatigue subscales of Profile of Mood States (POMS), which assesses short-term affective states. Respondents are instructed to rate how they feel “right now” [32], and a total score on each subscale is calculated. The subscales range from 0 to 28 (fatigue) and 0 to 32 (energy), with higher values indicating higher levels of fatigue and energy respectively. An evaluation has supported its use in experimental settings to measure short-term intensity of energy and fatigue [33].

#### Anxiety

There is support for acute exercise having effects on anxiety [12, 34], which is also commonly reported among ED patients [4]. State anxiety was measured using the 20-item state subscale of The State-Trait Anxiety Inventory (STAI-Y1) [25]. Respondents rate how they feel “right now” on a 4-point Likert Scale. A global score of state anxiety is calculated, ranging from 20–80, with higher scores corresponding to higher levels of state anxiety. STAI-Y1 has been extensively used in previous studies of acute exercise [34] and there is support for the STAI-Y1 being sensitive to change in response to acute aerobic exercise [14].

#### Stress

Since ED patients report increased sensitivity to stress [4] and physical activity is a stressor, we included a measure of subjective short-term stress. The Single Item Stress Question (SISQ) [35] was adapted by the researchers performing the study to measure stress “right now” instead of “these days”. Respondents rated their stress level on a 5-point Likert scale, ranging from 1 to 5, with higher ratings corresponding to higher stress.

The following measures were included at four time points: one immediately before the exercise, and three time points during the exercise:

#### Perceived exertion

Borg’s 6–20 rating of perceived exertion (RPE) [28] scale was used to assess exertion during exercise. It is a single-item scale ranging from 6 (not strenuous at all) to 20 (maximally strenuous).

#### Psychological discomfort

Subjective Units of Distress scale (SUDS) [30] was used to assess subjective psychological discomfort during exercise. Respondents were instructed to rate the level of psychological discomfort on a scale from 0 to 10, with higher values corresponding to more discomfort.

### Experimental conditions

The two experimental conditions, low and moderate exercise intensities, were conducted in a counterbalanced and randomly assigned cross-over design. The experimental condition order allocation sequence was determined through individual randomization from a computer-generated random order. This was made before data collection started, by a researcher involved in the study (JK). After the familiarization assessment was completed, each participant enrolled in the study was assigned her/his order of experimental conditions consecutively and equally. Researchers involved in the data collection had access to the password-protected document and could prepare for each experimental day accordingly. The exercise intensities were standardized across participants using their estimated maximal aerobic capacity (V̇O_2_ max) derived from the submaximal cardiorespiratory fitness tests. Intensities were based on classifications suggested by the American College of Sports Medicine [36], corresponding to 40 percent of V̇O2 max for low and 55 percent for moderate exercise intensity.

The participants performed the two exercise conditions at a similar time of day, starting either 8:30 or 10:30 in the morning, with at least one week between sessions. Blinding for condition or group was not practically possible. Hence, a detailed script for the researcher was integrated into the test protocol to maintain standardization and, thereby, internal validity. After receiving verbal instructions on the exercise bout, participants were asked to rate the level of perceived exertion (RPE) and feelings of psychological discomfort (SUDS) on visual analog scales (VAS), followed by individual adjustment of the seat and handlebars. Participants then performed the 22-min exercise on a cycle ergometer (model 839E, Monark, Varberg, Sweden), equipped with an automatic resistance adjustment feature, allowing participants to work out at their preferred RPM throughout the test while maintaining the intended rate of work. The exercise started with a 6-min warm-up phase, where the load was gradually increased from 1 min on 32 Watts, to 2 min on 50 percent, and then 2 min on 75 percent of the individualized work rate, before returning to 32 Watts for the last minute. Then followed 15 min on either low or moderate exercise intensity, which ended with a 1-min cool-down on 32 Watts. One of the researchers was present during the complete test to ensure correct intensity and length, and the conversation was kept to a minimum. The exercise duration was selected to provide enough time for participants to reach a circulatory and metabolic steady state but not lead to changes caused by prolonged exercise (such as dehydration or glycogen depletion). Previous research has also shown that significant changes in the primary outcome, fatigue, can be associated with exercise durations exceeding 20 min [18]. The first post-exercise measurements were followed by approximately 25 min of quiet rest in a seated position in an armchair with access to pillows and a blanket. During this quiet rest period talking was kept at a minimum, and participants were not allowed to use electronic devices but could choose from resting or reading magazines provided alternatively bring reading material of their own.

The participants were asked to rate their level of perceived exertion and psychological discomfort on visual analog scales during the exercise, at 5, 10, and 15 min on the 15-min exercise at low or moderate intensity. They completed questionnaires to measure fatigue, energy, anxiety, and stress at five time points: immediately before, immediately after, 30 min, 6 h, and 24 h after exercise. The initial three of these questionnaires were conducted during the experimental visit at GIH using tablets, while the latter two were digitally completed via links received via email. This extended measurement approach was designed to capture the immediate, short-term, and delayed psychological responses to exercise.

### Statistical analyses

Descriptive data from baseline measures were calculated for participant characteristics. Means and standard deviations were calculated for the primary and secondary study outcomes (fatigue, perceived exertion, psychological discomfort, energy, anxiety, stress) for each group for all time points in both conditions. Effects of the exercise conditions were assessed with repeated measures analysis of variance (RM-ANOVA) to determine the main effects of group (ED patients and controls), intensity (low and moderate), and time (four time points for perceived exertion and psychological discomfort (pre, 5, 10, and 15 min); five time points for fatigue, energy, anxiety, and stress (pre, post, 30 min post, 6 h post and 24 h post) and interaction effects for group x time, group x intensity, time x intensity, and group x time x intensity. One control participant was excluded from analyses on fatigue, stress, anxiety, and energy, and one ED participant was excluded from analyses on RPE and SUDS due to missing data. Significant interactions were followed up with paired comparisons, with Bonferroni corrections to reduce the Type 1-error risk. Effects sizes were calculated using generalized eta squared [37]. The level of significance (α) was set to 0.05 for all statistical tests. Assumption checks regarding sphericity, homogeneity of variances (Levene’s) as well as inspecting Q-Q-plots, were performed for all RM-ANOVAs. Mauchly’s Tests of Sphericity performed for all variables were all significant. When the Greenhouse–Geisser value was > 0.75, Huynh–Feldt correction was applied, and Greenhouse–Geisser correction was used for values < 0.75. There was overall an issue with skewness and homogeneity of variances for the variables fatigue, anxiety, stress, and psychological discomfort. After natural logarithm (ln) transformation of the variables anxiety and psychological discomfort, skewness was reduced, and none (anxiety) or few (psychological discomfort: 25%) of the Levene’s Test showed significant violations of the assumption of homogeneity of variance. Considering the issues with the assumptions of the RM-ANOVAs, significant results were verified with non-parametric tests. All data analyses were performed using Jamovi 2.3.28.

## Results

### Participant characteristics

Thirty participants with ED (SE-ICD-10; F43.8A) and thirty age- and sex-matched healthy controls completed the study. Independent samples t-tests showed that ED patients and control participants differed significantly in several health variables (see Table 1) and that the groups were similar in demographic variables.

**Table 1:** Baseline participant characteristics for the exhaustion disorder (ED) patients and the healthy controls

	**ED (*n* = 30)**	**Control (*n* = 30)**	***p*-value**
**Sex, women; *n* (%)**	28 (93.3)	28 (93.3)	1.00
**Age, years; mean (SD)**	46.3 (5.89)	45.8 (5.55)	0.719
**Highest education; *n* (%)**			0.539
Primary or upper-secondary	3 (10)	1 (3.33)	
Higher education	27 (90)	29 (96.67)	
**Employment status; *n* (%)**			0.151
Full time	24 (80)	28 (93.3)	
Part-time	3 (10)	1 (3.33)	
Unemployed	3 (10)	0 (0)	
Studying	0 (0)	1 (3.33)	
**Current sick leave; *n* (%)**	**23 (76.67)**	0 (0) *	** ≤ .001**
**Extend of sick leave; *n* (%)**		N/A	
25%	1 (3.33)	0 (0)	
50%	6 (20)	0 (0)	
75%	5 (16.67)	0 (0)	
100%	11 (36.67)	0 (0)	
**Self-reported ED duration, months; mean (SD)**	7.05 (5.98)	N/A	
**Self-reported peri- and menopausal symptoms; *n* (%)**	5 (21.7)	5 (18.5)	0.777
**Body Mass Index (BMI); mean (SD)**	26.1 (5.39)	25.2 (3.98)	0.470
**Cardiorespiratory fitness (VO2; l/min); mean (SD)**	**2.41 (0.48)**	2.85 (0.42) *	** ≤ .001**
**Cardiorespiratory fitness (VO2; ml/min/kg); mean (SD)**	**34.1 (7.93)**	40.6 (7.04) *	** ≤ .001**
**Physical activity (SGPALS); *n* (%)**		*	** ≤ .001**
Almost completely inactive	0 (0)	0 (0)	N/A
Moderately active	**17 (56.67)**	4 (13.33) *	** ≤ .001**
Highly active	**11 (36.67)**	19 (63.33) *	**0.039**
Vigorously active	2 (6.67)	7 (23.33)	0.073
**Psychiatric diagnosis; *n* (%)**		*	** ≤ .001**
Depression	**4 (13.33)**	0 (0) *	**0.038**
Anxiety disorders	**5 (16.67)**	0 (0) *	**0.019**
**Medication; *n* (%)**		*	** ≤ .001**
Antidepressants	**15 (50)**	3 (10) *	** ≤ .001**
Anxiolytics	2 (6.67)	0 (0)	0.155
Sleeping medication	2 (6.67)	0 (0)	0.150
**Exhaustion disorder (KEDS; range 0–45); mean (SD)**	**32 (5.69)**	5.6 (3.2) *	** ≤ .001**
**Burnout (SMBM-12; range 1–7); mean (SD)**	**5.35 (0.95)**	1.80 (0.61) *	** ≤ .001**
**Trait anxiety (STAI-T; range 20–80); mean (SD)**	**53.2 (8.39)**	36.1 (5.24) *	** ≤ .001**
**Depression (PHQ-9; range 0–27); mean (SD)**	**11.9 (4.6)**	1.63 (1.25) *	** ≤ .001**
**Sleep quality (PSQI; range 0–21); mean (SD)**	**8.4 (2.75)**	4.14 (1.51) *	** ≤ .001**

^*^indicates differences between groups at *p* < 0.05

### Changes in fatigue, energy, anxiety, and stress after exercise

#### Fatigue

**Fig. 2:**
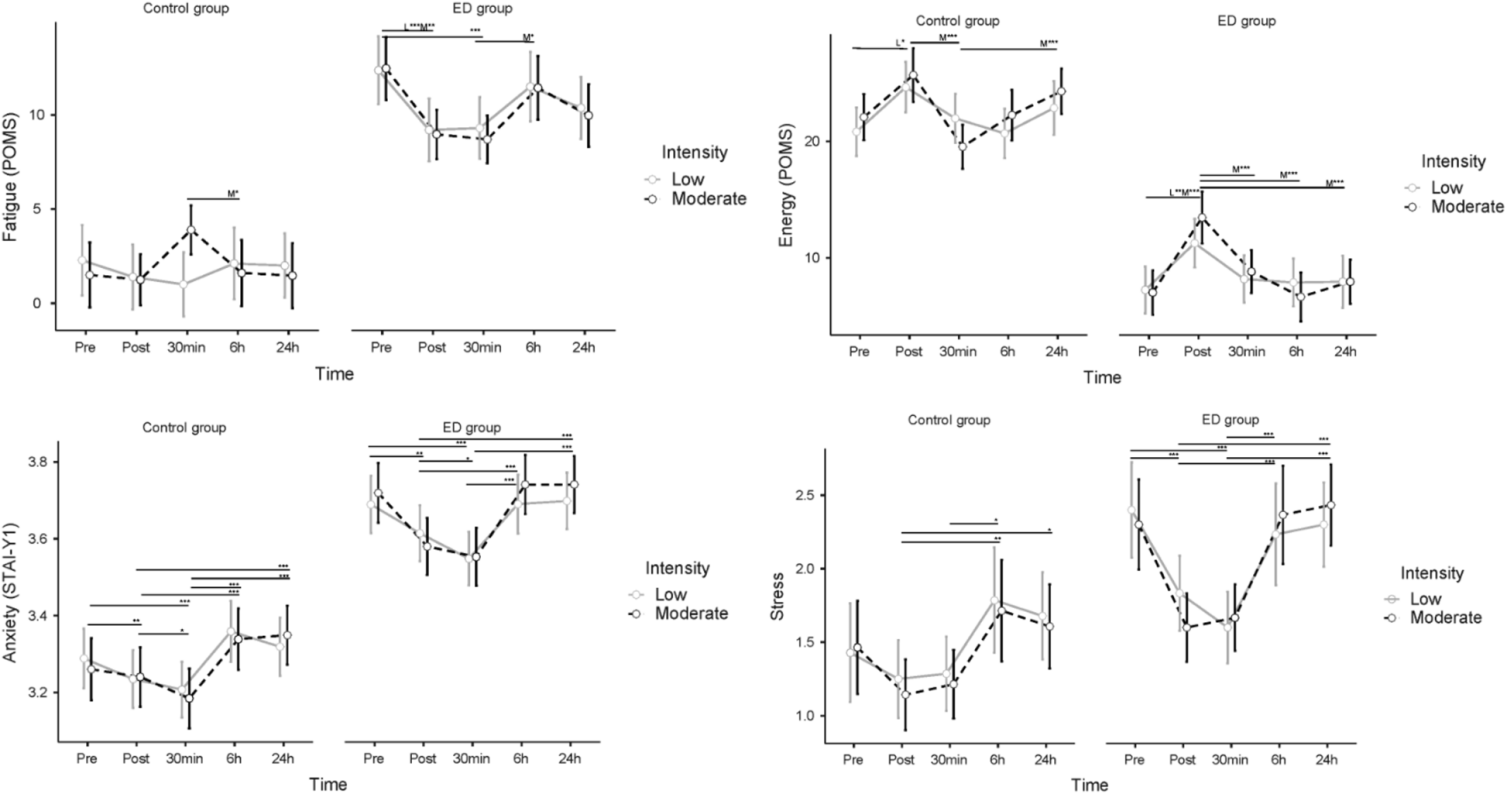
Changes over time in fatigue (POMS, range 0–28), energy (POMS, range 0–32), stress (range 1–5), and ln-transformed anxiety (lnSTAI-Y1). No significant differences were found between pre- and 24-h post-exercise measurements for any outcome. There were significant group differences for all outcomes. * *p* ≤ 0.05, ** *p* ≤ 0.01, *** p ≤ 0.001. M = moderate-intensity exercise effect was significant. L = low-intensity exercise effect was significant

For fatigue, there was a significant interaction effect of time x intensity x group (η^2^_G_ = 0.008). There were significant main effects of group (η^2^_G_ = 0.49) and time (η^2^_G_ = 0.024) but not of intensity (see Fig. 2 and Table 2). Post hoc tests on the significant time x intensity x group interaction effect (see Table S3, Additional file 1) revealed significant group differences on all time points in fatigue rating, i.e. the ED group reported higher levels of fatigue than the control group throughout all time points. The ED group had a reduction in fatigue immediately after exercise (post) compared to pre-exercise, which was sustained thirty minutes later (30 min post). This pattern was the same across the two intensities. The control group did not have the same direct exercise effect as the ED group. On the contrary, there were no differences between any measure points for the control group at low intensity, while at moderate intensity, the only significant difference between time points was higher fatigue levels 30 min post-exercise compared to immediately post-exercise. There were no significant differences between the pre-measure and the measures 6 h or 24 h post-exercise for either group.

**Table 2:** Main and interaction effects of group (exhaustion disorder (ED) and control group), time (pre-, post-, 30 min post-, 6 h post-, and 24 h post-exercise), and intensity (low and moderate) in RM-ANOVA

Outcome	Group (G)	Time (T)	Intensity (I)	T x G	I x G	T x I	T x I x G
	η^2^_G_ (*p*)	η^2^_G_ (*p*)	η^2^_G_ (*p*)	η^2^_G_ (*p*)	η^2^_G_ (*p*)	η^2^_G_ (*p*)	η^2^_G_ (*p*)
Fatigue	**0.49 (< .001)**	**0.024 (< .001)**	0.00 (0.92)	**0.025 (< .001)**	0.001 (0.44)	0.005 (0.13)	**0.008 (0.022)**
Energy	**0.61 (< .001)**	**0.086 (< .001)**	0.001 (0.15)	**0.012 (0.014)**	0.00 (0.61)	**0.006 (0.013)**	**0.009 (< .001)**
lnAnxiety	**0.47 (< .001)**	**0.089 (< .001)**	0.00 (0.54)	(0.005 (0.36)	0.001 (0.17)	0.002 (0.36)	0.002 (0.30)
Stress	**0.14 (< .001)**	**0.10 (< .001)**	0.00 (0.62)	**0.013 (0.034)**	0.00 (0.62)	0.002 (0.53)	0.003 (0.49)
RPE	**0.11 (< .001)**	**0.82 (< .001)**	**0.23 (< .001)**	0.013 (0.08)	0.005 (0.13)	**0.089 (< .001)**	0.001 (0.42)
lnSUDS	**0.20 (< .001)**	0.008 (0.11)	0.00 (0.81)	0.002 (0.59)	0.00 (0.71)	0.00 (0.95)	0.00 (0.70)

*RPE* Rating of perceived exertion*, lnSUDS* Subjective Units of Distress Scale (psychological discomfort)

#### Energy

There was a significant interaction effect of time x intensity x group on energy (η^2^_G_ = 0.009), as well as significant main effects of group (η^2^_G_ = 0.61) and time (η^2^_G_ = 0.086) but not of intensity (see Fig. 2 and Table 2). Post hoc tests on the significant time x intensity x group interaction effect (see Table S4, Additional file 1) showed significant group differences on all time points in energy rating, i.e. the ED group reported lower energy than the control group throughout all corresponding time points. The ED group had a significant energy increase directly after exercises (post) on both intensities, while for the control group, this effect was significant only after the low-intensity exercise. Both groups had a significant decrease in energy 30 min after exercise compared to post-exercise at moderate, but not low, intensity. There were no significant differences in either group between the pre-measures and the measures 6 h or 24 h post-exercise.

#### Anxiety

There were significant main effects of group (η^2^_G_ = 0.47) and time (η^2^_G_ = 0.089), but not of intensity, on anxiety (see Fig. 2 and Table 2). There were no interaction effects. The ED group reported significantly higher anxiety than the control group. Post hoc tests for the main effect of time (see Table S5, Additional file 1) revealed significantly lower anxiety directly after exercise (post) compared to pre-exercise. Anxiety declined even further 30 min after exercise, reflected in significantly lower anxiety levels 30 min post-exercise compared to post-exercise. There were no significant differences between the pre-measure and the measures 6 h or 24 h post-exercise.

#### Stress

There was a significant interaction effect of time x group on stress (η^2^_G_ = 0.013), as well as significant main effects of group (η^2^_G_ = 0.14) and time (η^2^_G_ = 0.10) but not of intensity (see Fig. 2 and Table 2). Post hoc tests on the time x group interaction effect (see Table S6, Additional file 1) revealed that the ED group reported significantly higher stress levels than the control group on all corresponding time points except at 30 min and 6 h after exercise. Additional group differences were that the ED group, but not the control group, experienced significantly lower stress levels immediately after exercise (post) compared to pre-exercise, which was maintained 30 min after exercise. There were no significant differences between the pre-measure and the measures 6 h or 24 h post, however, both groups displayed significantly higher stress levels 6 and 24 h after exercise compared to directly after (post) exercise.

### Changes during exercise in perceived exertion and perceived psychological discomfort

#### Perceived exertion

**Fig. 3:**
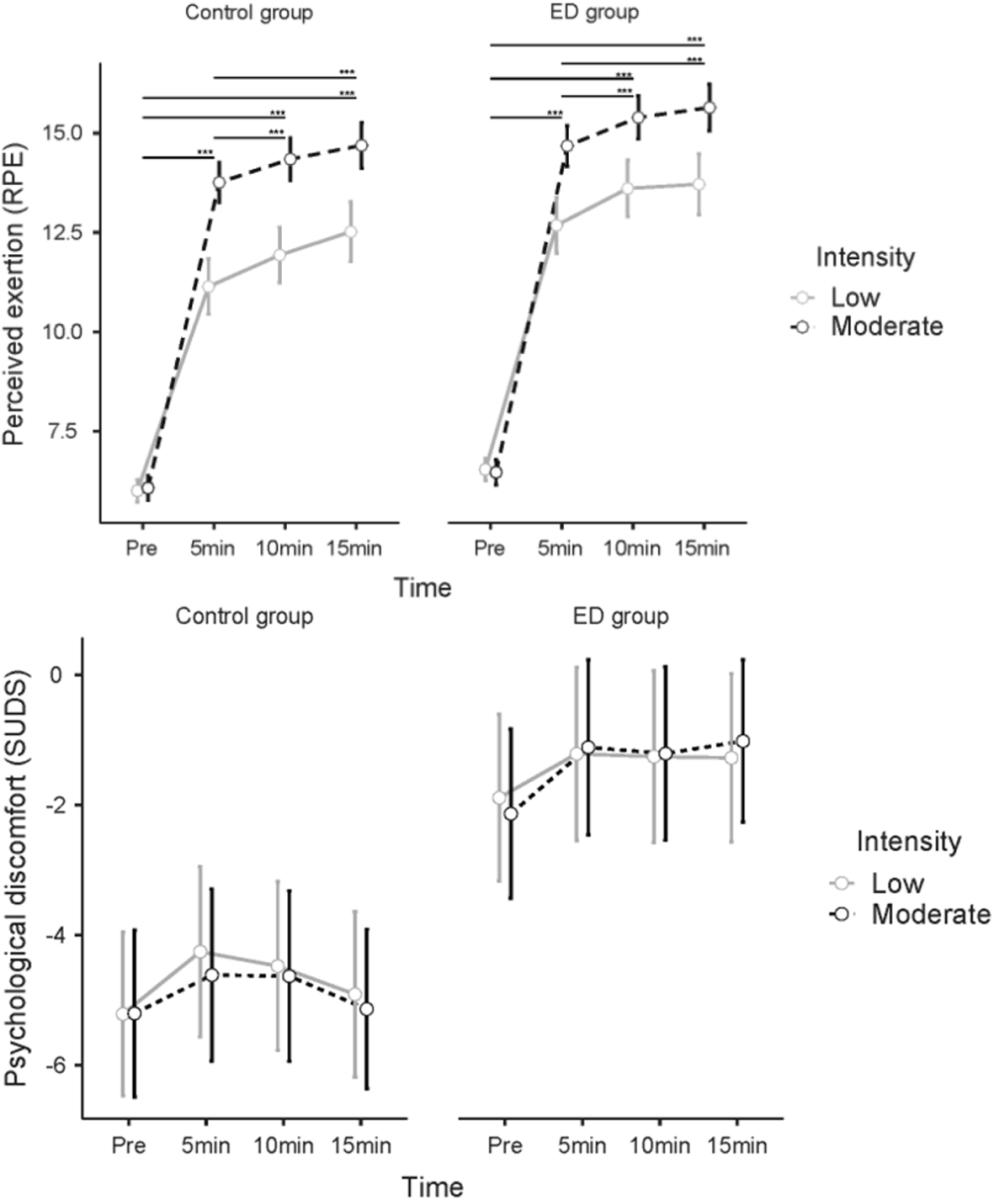
Change over time in perceived exertion (RPE, range 6–20) and ln-transformed psychological discomfort (lnSUDS) during exercise. No significant differences were found between pre- and 24-h post-exercise measurements for any outcome. There were significant group differences for all outcomes. * *p* ≤ 0.05, ** *p* ≤ 0.01, *** p ≤ 0.001. M = moderate-intensity exercise effect was significant. L = low-intensity exercise effect was significant

Regarding perceived exertion (RPE), there was a significant interaction effect of time x intensity (η^2^_G_ = 0.089), and significant main effects of group (η^2^_G_ = 0.11), time (η^2^_G_ = 0.82), and intensity (η^2^_G_ = 0.23) (see Fig. 3 and Table 2). The ED group reported a significantly higher RPE than the control group, showing that the exercise bouts were perceived as more strenuous for ED patients than for healthy controls. Post hoc tests on the significant time x intensity interaction effect showed that RPE increased significantly throughout all consecutive time points during the exercise bouts except from 10 to 15 min at both intensities. The participants reported significantly higher RPE at moderate than at low intensity. For more information on the post hoc tests, see Table S2 in Additional file 1.

#### Psychological discomfort

There was a significant main effect of group (η^2^_G_ = 0.20) on psychological discomfort (see Fig. 3 and Table 2). The ED group experienced more discomfort than the control group. There were no main effects of time or intensity and no interaction effects regarding discomfort.

### Drop-out

More eligible participants in the ED group (n = 16) dropped out than in the control group (n = 9) (see Fig. 1). The difference can be attributed to more ED patients declining before the study phase with the two experimental exercise visits. 118 out of the 120 exercise sessions were completed. Two participants in the ED group terminated the moderate-intensity exercise before completion. This occurred 10 min and 19 min, respectively, into the exercise session, both due to the participants perceiving the feeling of exertion as too high (18 and 20 RPE, respectively). Data from the participant who stopped after 19 min was included as the session was almost fully completed, and the control analysis confirmed that it did not affect the analyses.

## Discussion

To our knowledge, this study is the first to investigate the psychological responses to acute exercise in patients with stress-induced exhaustion disorder (ED). Our primary aim was to compare these responses between ED patients and healthy controls, facilitating an understanding of typical exercise reactions in the ED group in contrast to non-clinical individuals. Our findings show that a short bout of aerobic exercise can alleviate symptoms experienced by ED patients, consistent with previous research on the effects of exercise on symptoms in patients with depression and anxiety disorders [13–15]. As expected, ED patients, compared to healthy controls, reported higher general levels of fatigue, anxiety, and stress, and lower levels of energy, reflecting the common symptoms of ED. The exercise effects in ED patients differed from those in healthy controls in several ways. ED patients perceived the exercise as more strenuous but experienced greater reductions in fatigue and stress immediately after exercise, with effects also sustained after a 30-min rest. Additionally, ED patients showed a more pronounced energy increase post-exercise. Furthermore, we aimed to explore whether psychological responses varied between low and moderate exercise intensities. We found that ED patients exhibited similar psychological responses to both intensities.

During the exercise sessions, the ED patients experienced the activity as more strenuous than the healthy controls. This was expected given that mentally fatigued individuals rate exercise as more strenuous [38] and that marked physical weakness is a symptom of ED (ICD-10-SE). Hence, the higher self-reported exertion in ED patients may be attributed to central fatigue [39], an issue reported in studies on similar disorders [5, 40], rather than solely the physiological processes in exercise seen in overall healthy individuals. Central nervous system-related fatigue affects exercise performance together with peripheral fatigue also in healthy individuals but can be more pronounced in disorders such as ME/CFS and depression [5]. Previous studies in ME/CFS patients have shown higher perceived exertion ratings in relation to heart rate during exercise despite no defect in neuromuscular function, which has been attributed in part to central fatigue [40].

Interestingly, the elevation of exertion did not correspond with that of psychological discomfort. Although ED patients reported greater overall psychological discomfort, they did not experience a higher increase in discomfort compared to healthy controls. This is consistent with previous research in women with ED, showing higher overall SUDS ratings but no difference in psychological distress response compared to a control group when exposed to a mental stressor [41]. Hence, ED patients seem to respond similarly to mental and physical stressors in terms of psychological discomfort. As our study measured both perceived exertion and psychological discomfort during the exercise, the complexity of how exercise is experienced was highlighted. The notion that more strenuous physical activity does not necessarily correspond to increased psychological discomfort has support from previous research, which shows that affective displeasure does not correlate linearly with exercise intensity but instead plateaus at higher intensities above the ventilatory threshold [12], typically correlating to RPE ratings around 13 [42].

Despite the exercise being perceived as more strenuous by the ED group compared to non-clinical individuals, it elicited more beneficial psychological effects post-exercise. Previous research performed primarily on healthy individuals has found that acute exercise consistently increases energy [18]. However, results on fatigue are more heterogeneous, showing that fatigue reduction occurs when baseline fatigue is normal or high and when energy increase post-exercise is substantial [18]. The reductions in fatigue and increased energy in ED patients are hence consistent with these results. A study on college students with elevated fatigue [43] found similar reductions in fatigue after exercise at 50% of V̇O_2_ max, resembling our study’s moderate-intensity exercise, but not at 75% of V̇O_2_ max. The fatigue reductions in the ED group contrast with the elevated fatigue in ME/CFS patients post-exercise [16], a condition similar to ED characterized by persistent fatigue. This highlights differences between the two conditions. While ED patients experienced lowered fatigue post-exercise, controls did not, likely due to a floor effect, as their pre-exercise fatigue levels were already low (see Table S8, Additional file 1). In previous acute exercise studies showing fatigue-reducing effects, pre-exercise POMS fatigue scores averaged 6.42 [18], allowing for noticeable reductions.

Conversely, the control group showed increased fatigue 30 min after moderate-intensity exercise. Typically, post-exercise fatigue increases occur when the baseline fatigue level is low [18], as in this study’s control group, but are otherwise also associated with high-intensity and longer-duration exercise [18]. Sustained reductions in fatigue have been observed in both healthy individuals [44] and those with substance abuse [45]. However, a difference from these studies is that participants generally experienced an immediate fatigue-reducing effect, which was not observed in the control group of this study.

Both groups experienced elevated energy post-exercise, in line with previous research [18]. For ED patients, this effect was significant at both intensities, while for healthy controls, it was significant only at low intensity, though approaching significance at moderate intensity (*p* = 0.089). The lack of a pronounced energy increase from moderate-intensity exercise in the control group might contribute to the explanation of the delayed fatigue observed 30 min post-exercise, since energy has been shown to moderate changes in fatigue [18].

Besides a significantly more pronounced energy drop from post-exercise to 30 min after exercise at moderate intensity for both groups, and elevated fatigue in the control group 30 min after moderate-intensity exercise, the short exercise bouts did not produce significantly different psychological responses based on intensity in either group. Previous research on intensity effects on affective states shows mixed results [12]. Many studies report no intensity effects, while others have found varied or negative effects on higher exercise intensities, particularly for energy and fatigue. However, a meta-analysis suggested greater anxiety-reducing effects at higher intensities due to the exposure, habituation, and reappraisal of bodily sensations similar to anxiety reactions (e.g., elevated heart rate, elevated breathing rate) [34]. Consequently, low-intensity exercise usually does not produce beneficial changes in fatigue, energy, and anxiety, as measured in this study. A more mechanistic study design could shed light on the mechanisms underlying these patterns in ED patients.

Previous research generally shows small reductions in state anxiety [34], and an anxiety-reducing effect of the exercise was also observed in this study. The time x group interaction only approached significance (*p* = 0.077), making it uncertain how the two groups differed in their responses, but importantly, the ED group did not show increased anxiety after exercise (see Table S8, Additional file 1). Interestingly, anxiety levels decreased further during the 30-min rest period following exercise, an effect not observed in previous studies, such as a meta-analysis including the same anxiety measure used here [34], or a recent study on depression patients that included a post-exercise rest period [46]. A partial explanation could be that the rest period was particularly valued by the ED patients due to their symptoms of exhaustion. Although not a part of the data collection protocol, anecdotal data indicated that many ED patients viewed the mandatory rest as a welcome treat. Additionally, the self-selected component of the quiet rest, where participants could choose to rest, sleep, read provided magazines, or bring their own reading material, may have contributed to the rest being viewed as an overall positive experience. The stress response curve was similar to that of anxiety, indicating an overlap between the two constructs. A recent meta-analysis on stress reactivity to a mental stressor found no reliable changes in self-reported stress after acute exercise [47], hence again highlighting the need to study mechanisms underlying responses.

Significant group differences were observed in exercise-related measures (see Table 1), with the ED group reporting lower physical activity levels over the past year and exhibiting lower fitness levels compared to the control group. Baseline physical activity and fitness have been shown to mediate exercise responses, although the data is primarily based on non-clinical populations. While research on the impact of physical activity levels on acute exercise effects regarding fatigue and energy is limited [18], one study found that regular exercisers showed significant changes in energy and fatigue, while non-exercisers did not [48]. A meta-analysis found that sedentary individuals experienced the largest reductions in anxiety following exercise [34]. Additionally, both fit and unfit participants reported positive affective responses to low-intensity exercise compared to high-intensity exercise [49]. Overall, the available data is heterogeneous, suggesting that the relationship between physical activity, fitness, and exercise responses is complex and potentially confounded by unreported factors. While we cannot confirm if the ED patients in this study fully represent the broader ED population in these aspects, a comparison of the cardiorespiratory fitness level in our ED group (34.1; see Table 1) with that of ED patients in a randomized trial comparing exercise training, cognitive training, and a control condition shows comparable baseline fitness levels (34.66–35.96) [50].

We believe that the measures 6 and 24 h after exercise were important, as clinical observations suggest that concerns about delayed post-exercise fatigue and reduced energy often discourage physical activity and exercise in individuals with ED. Recent qualitative research [4] has supported this. Given that the psychological effects of acute exercise are transient, with positive activated affect like energy typically lasting up to 30 min [51], we did not expect improvements later in the day or the next morning. However, we considered potential delayed negative psychological effects, similar to those reported by ME/CFS patients [16], especially since ED patients have reported such expectations [4]. Contrary to these assumptions, neither group showed increased fatigue, anxiety, stress, or decreased energy later in the day or the following morning compared to baseline, which is encouraging findings. It is important to note that this only concerns the short-term effects of single exercise sessions, and no conclusions can be drawn about the effects of repeated exercise. One factor potentially influencing the delayed ratings is the quiet rest post-exercise. It is possible that a rest period after exercise affected the ED group more since lack of recovery, i.e., “psychophysiological unwinding after effort expenditure” [52], is central in the link between exposure to stressors and negative effects of stress. Hence, the delayed measurements show how exercise followed by 30 min of quiet rest affected the participants, not solely exercise.

Fatigue, being a central clinical characteristic of ED [3, 4], can be defined as a “persistent sense of physical, emotional, and/or cognitive tiredness or exhaustion” [31] and implies a lack of ability to initialize and maintain mental and physical tasks that require effort and self-motivation. This has implications for the real-world transferability of the high compliance and low dropout rates in the exercise sessions, as participants had support in initiating the exercise, and researchers were present throughout the sessions. Social support is an important factor for ED patients with less exercise experience in establishing an exercise routine [53]. Previous research shows that mental fatigue affects the choice of sedentary activities over physical activities, with the only exception being low intensities [54]. So, although our results have positive immediate effects on symptoms, the effort required to initiate and maintain short exercise sessions might be a considerable obstacle. This must be addressed in intervention design.

There is limited data on how generalizable acute exercise studies are to long-term exercise. Thus, our study does not answer how ED patients would react psychologically to regular exercise, warranting further studies. Acute exercise effects can however increase the chance of continuous exercise adherence, with previous research showing that lower levels of fatigue post-exercise correlate with more frequent exercise months later [55]. The beneficial effects of single exercise sessions observed here might thus potentially increase compliance with exercise in ED patients, by enhancing external motivation and limiting negative expectations of exercise consequences.

### Potential limitations

One factor not manipulated in the study was the duration of exercise, which is another aspect influencing the psychological responses to exercise. A meta-analysis on acute exercise effects on fatigue and energy [31] showed that while exercise consistently increases feelings of energy, fatigue increases with longer duration, specifically beyond 20 min. However, a study comparing various combinations of durations of acute exercise (5–60 min) and subsequent rest periods showed no difference in fatigue ratings [44]. The exercise bouts in that study produced mean RPE ratings between 9.7 and 11.9, hence comparable to the low-intensity exercise in our study (see Table S7, Additional file 1). But considering that fatigue is central to ED, it might be that longer durations of exercise beyond 20 min affect this group more than many other populations. This highlights that the combination of intensity and duration affects the psychological responses and must be considered jointly.

Estimation of aerobic capacity was performed using an indirect method. As such, it contains errors larger than those related to direct or maximal test methods. However, maximal tests were deemed unsuitable for the ED group and any comparison to the controls could have been attributed to differences in ability to reach maximal effort. Nonetheless, the errors related to the submaximal, indirect test may have resulted in over- or underestimations, potentially causing participants in both groups to work at a higher or lower work rate than the intended 40% and 55%. We regard this as a random error that increases the variance and potentially results in an underestimation of real changes or differences, but not creating systematic group or time differences. Concerning blood pressure, we have no record on which arm it was measured, so there may have been variations. Since these measures were used solely for exclusion purposes and no participant was approaching the exclusion limit, we consider this a minor limitation. Another limitation regarding internal validity is that some participants may have slept during the resting period after the exercise bouts, which was not systematically registered.

We did not include a measure of positive affect during exercise, which could have given a more comprehensive understanding of the psychological responses [12]. Future studies should address this. However, one strength of our study is the inclusion of some psychological measures during exercise, which is often overlooked in acute exercise research. Since psychological responses are dynamic and not linear, this, combined with delayed measurements, provides a more complete picture of the psychological responses to exercise in ED patients.

The sample consisted primarily of women, with two men in each group. This limits the generalizability of the results to men. Gender differences in psychological responses to acute exercise are understudied. Still, one study [56] on young adults found such differences, with women showing greater improvements than men in fatigue and energy after 30 min of vigorous aerobic exercise. Generally, those with worse baseline values for variables such as anxiety [34] and energy level [51] experience significant beneficial effects of the exercise. Since women reported higher baseline problems with these variables, the observed differences might be due to the larger potential for benefits rather than to gender differences.

Experimental studies in a laboratory environment always have the issue of generalizability or external validity. Factors that could have contributed to negative experiences during the exercise (e.g. level of exertion and psychological discomfort) include that the exercise took place in a windowless room without distractions such as music or scenery. Exercising outdoors vs. indoors has been proposed to elicit lower RPE, and listening to music has also been shown to have beneficial effects on RPE [57]. The presence of a researcher during exercise might have affected the participants. Non-systematic observations indicated that some ED patients felt nervous about exercising, especially at moderate intensity, but felt safer in the controlled situation with a researcher present.

Another limitation of this study is the issue of multiple testing. While Bonferroni corrections were applied to control for Type I errors, the large number of tests still introduces some risk of false findings. However, given that the results generally pointed in the same direction, the likelihood of Type I errors is likely reduced. On the other hand, using a conservative correction may have limited the ability to detect smaller effects, particularly in the control group, which could be a source of Type II error. Despite this, the clear direction of the results supports the overall validity of the findings.

However, a strength of the study is the comparison of the ED patients to a healthy control group, which allowed reliable conclusions about the psychological responses to exercise specific to ED patients. This makes adjusting the exercise recommendations easier. Additionally, counterbalancing exercise session order based on intensity enhances the reliability of conclusions about intensity effects, avoiding potential novelty effects on psychological discomfort, stress, and anxiety.

Lastly, we had concerns that only ED patients with low symptom levels and limited impairment in important areas of functioning would participate in the study. This was, however, not substantiated, as demonstrated in the baseline exhaustion ratings and sickness absence (see Table 1). The large interest shown by individuals with an ED diagnosis in participating in the study indicates a large interest among them in exercise as an intervention.

### Further research

This study aimed to shed some initial light on how acute exercise affects patients with ED, but several questions remain unanswered. Future studies could include exercise with different durations and higher exercise intensities, as well as explore the influence of different durations of rest post-exercise. The focus was on psychological responses to acute exercise, without exploring underlying processes that govern these responses in ED patients. Studies with a mechanistic focus can contribute to understanding the processes that underlie the psychological changes during and after exercise in ED patients. Research with a focus on susceptibility profiles is also needed, enabling more tailored exercise programs for the different needs of different ED patients. Potential moderators could include symptom severity, exercise level, fitness level, and BMI. While this study focused on aerobic exercise, other types of training, such as strength training, could also have beneficial psychological effects, and warrant further research.

## Conclusions

This study shows the specific psychological responses of ED patients to acute exercise, a condition characterized by persistent fatigue due to chronic psychosocial stress. ED patients showed greater reductions in fatigue and stress and increases in energy post-exercise compared to healthy controls, with both groups experiencing anxiety reductions. These benefits were observed at both low and moderate intensities, suggesting that ED patients may have psychological benefits from exercise regardless of intensity. Despite perceiving exercise as more strenuous, ED patients reported no increased psychological discomfort, indicating central fatigue drives their exertion. No delayed negative effects were observed, which may alleviate concerns about exercise-induced fatigue. These findings enhance our understanding of how ED patients uniquely respond to exercise and can guide intervention design that enhances long-term exercise adherence [12].

## Supplementary Information

Additional file 1

Additional file 2

## Data Availability

The data that was analyzed or produced in this study is not openly accessible due to Swedish legislation (the Swedish Ethical Review Act: 2003:460) but the authors can provide access upon reasonable request. For such inquiries, please contact VB.

## References

[1] Försäkringskassan. Sjukfrånvaro i psykiatriska diagnoser - En registerstudie av Sveriges arbetande befolkning i åldern 20–69 år. Analys och prognos; 2020 p. 108. Report No.: Socialförsäkringsrapport 2020:8. Available from: https://www.forsakringskassan.se/download/18.7fc616c01814e179a9f329/1656660446139/sjukfranvaro-i-psykiatriska-diagnoser-socialforsakringsrapport-2020-8.pdf. Accessed 29 Aug 2024.

[2] Socialstyrelsen. Utmattningssyndrom: stressrelaterad psykisk ohälsa. Stockholm: Socialstyrelsen; 2003.

[3] Grossi G, Perski A, Osika W, Savic I. Stress-related exhaustion disorder - clinical manifestation of burnout? A review of assessment methods, sleep impairments, cognitive disturbances, and neuro-biological and physiological changes in clinical burnout. Scand J Psychol. 2015;56(6):626–36. 26496458 10.1111/sjop.12251

[4] Lindsäter E, Svärdman F, Rosquist P, Wallert J, Ivanova E, Lekander M, et al. Characterization of exhaustion disorder and identification of outcomes that matter to patients: Qualitative content analysis of a Swedish national online survey. Stress Health. 2023;39(4):813–27. 36645034 10.1002/smi.3224

[5] Leavitt VM, DeLuca J. Central fatigue: issues related to cognition, mood and behavior, and psychiatric diagnoses. PM R. 2010;2(5):332–7. 20656614 10.1016/j.pmrj.2010.03.027

[6] Hassard J, Teoh KRH, Visockaite G, Dewe P, Cox T. The cost of work-related stress to society: A systematic review. J Occup Health Psychol. 2018;23(1):1–17. 28358567 10.1037/ocp0000069

[7] Lindsäter E, Svärdman F, Wallert J, Ivanova E, Söderholm A, Fondberg R, et al. Exhaustion disorder: a scoping review of research on a recently introduced stress-related diagnosis. BJPsych Open. 2022;8(5):e159. 36458830 10.1192/bjo.2022.559 PMC9438479

[8] Singh B, Olds T, Curtis R, Dumuid D, Virgara R, Watson A, et al. Effectiveness of physical activity interventions for improving depression, anxiety and distress: an overview of systematic reviews. Br J Sports Med. 2023;57(18):1203–9. 36796860 10.1136/bjsports-2022-106195 PMC10579187

[9] Ochentel O, Humphrey C, Pfeifer K. Efficacy of exercise therapy in persons with burnout: a systematic review and meta-analysis. J Sports Sci Med. 2018;17(3):475–84. 30116121 PMC6090391

[10] Lindegård A, Jonsdottir IH, Börjesson M, Lindwall M, Gerber M. Changes in mental health in compliers and non-compliers with physical activity recommendations in patients with stress-related exhaustion. BMC Psychiatry. 2015;4(15):272. 10.1186/s12888-015-0642-3 PMC4632342 26530329

[11] Eskilsson T, SlungaJärvholm L, MalmbergGavelin H, Stigsdotter Neely A, Boraxbekk CJ. Aerobic training for improved memory in patients with stress-related exhaustion: a randomized controlled trial. BMC Psychiatry. 2017;17(1):322. 28865430 10.1186/s12888-017-1457-1 PMC5581420

[12] Ekkekakis P, Parfitt G, Petruzzello SJ. The pleasure and displeasure people feel when they exercise at different intensities: decennial update and progress towards a tripartite rationale for exercise intensity prescription. Sports Med. 2011;41(8):641–71. 21780850 10.2165/11590680-000000000-00000

[13] Meyer JD, Koltyn KF, Stegner AJ, Kim JS, Cook DB. Influence of exercise intensity for improving depressed mood in depression: a dose-response study. Behav Ther. 2016;47(4):527–37. 27423168 10.1016/j.beth.2016.04.003

[14] Herring MP, Monroe DC, Gordon BR, Hallgren M, Campbell MJ. Acute exercise effects among young adults with analogue generalized anxiety disorder. Med Sci Sports Exerc. 2019;51(5):962–9. 30531490 10.1249/MSS.0000000000001860 PMC7218918

[15] Ströhle A, Graetz B, Scheel M, Wittmann A, Feller C, Heinz A, et al. The acute antipanic and anxiolytic activity of aerobic exercise in patients with panic disorder and healthy control subjects. J Psychiatr Res. 2009;43(12):1013–7. 19289240 10.1016/j.jpsychires.2009.02.004

[16] Loy BD, O’Connor PJ, Dishman RK. Effect of acute exercise on fatigue in people with ME/CFS/SEID: a meta-analysis. Med Sci Sports Exerc. 2016;48(10):2003–12. 27187093 10.1249/MSS.0000000000000990 PMC5026555

[17] Maroti D, Molander P, Bileviciute-Ljungar I. Differences in alexithymia and emotional awareness in exhaustion syndrome and chronic fatigue syndrome. Scand J Psychol. 2017;58(1):52–61. 27686801 10.1111/sjop.12332

[18] Loy BD, O’Connor PJ, Dishman RK. The effect of a single bout of exercise on energy and fatigue states: a systematic review and meta-analysis. Fatigue. 2013;1(4):223–42.

[19] Besèr A, Sorjonen K, Wahlberg K, Peterson U, Nygren A, Asberg M. Construction and evaluation of a self rating scale for stress-induced exhaustion disorder, the Karolinska exhaustion disorder scale. Scand J Psychol. 2014;55(1):72–82. 24236500 10.1111/sjop.12088 PMC4235404

[20] Sheehan DV, Lecrubier Y, Sheehan KH, Amorim P, Janavs J, Weiller E, et al. The Mini-International Neuropsychiatric Interview (MINI): the development and validation of a structured diagnostic psychiatric interview for DSM-IV and ICD-10. J Clin Psychiatry. 1998;59 Suppl 20:22–33. quiz 34-57. 9881538

[21] World Health Organization. ICD-11: International classification of diseases. 2019. Available from: https://icd.who.int/. Accessed 29 Aug 2024.

[22] Åsberg M, editor. Utmattningssyndrom. 1st ed. Gothia Kompetens: Stockholm; 2024.

[23] Saltin B, Grimby G. Physiological analysis of middle-aged and old former athletes. Comparison with still active athletes of the same ages. Circulation. 1968;38(6):1104–15. 5721960 10.1161/01.cir.38.6.1104

[24] Almén N, Jansson B. The reliability and factorial validity of different versions of the Shirom-Melamed Burnout Measure/Questionnaire and normative data for a general Swedish sample. Int J Stress Manag. 2021;28(4):314–25.

[25] Spielberger CD, Gorsuch RL, Lushene RE. The State-Trait Anxiety Inventory: Testmanual. Palo Alto: Consulting Psychologist Press; 1970.

[26] Kroenke K, Spitzer RL, Williams JB. The PHQ-9: validity of a brief depression severity measure. J Gen Intern Med. 2001;16(9):606–13. 11556941 10.1046/j.1525-1497.2001.016009606.x PMC1495268

[27] Buysse DJ, Reynolds CF 3rd, Monk TH, Berman SR, Kupfer DJ. The Pittsburgh Sleep Quality Index: a new instrument for psychiatric practice and research. Psychiatry Res. 1989;28(2):193–213. 2748771 10.1016/0165-1781(89)90047-4

[28] Björkman F, Ekblom-Bak E, Ekblom Ö, Ekblom B. Validity of the revised Ekblom Bak cycle ergometer test in adults. Eur J Appl Physiol. 2016;116(9):1627–38. 27311582 10.1007/s00421-016-3412-0 PMC4983286

[29] Borg G, Ljunggren G, Ceci R. The increase of perceived exertion, aches and pain in the legs, heart rate and blood lactate during exercise on a bicycle ergometer. Eur J Appl Physiol Occup Physiol. 1985;54(4):343–9. 4065121 10.1007/BF02337176

[30] Tanner BA. Validity of global physical and emotional SUDS. Appl Psychophysiol Biofeedback. 2012;37(1):31–4. 22038278 10.1007/s10484-011-9174-x

[31] Loy BD, Cameron MH, O’Connor PJ. Perceived fatigue and energy are independent unipolar states: Supporting evidence. Med Hypotheses. 2018;113:46–51. 29523293 10.1016/j.mehy.2018.02.014 PMC5846196

[32] McNair DM, Lorr M, Droppleman L. Profile of Mood States questionnaire. San Diego: EDITS; 1981.

[33] O’Connor PJ. Evaluation of four highly cited energy and fatigue mood measures. J Psychosom Res. 2004;57(5):435–41. 15581646 10.1016/j.jpsychores.2003.12.006

[34] Ensari I, Greenlee TA, Motl RW, Petruzzello SJ. Meta-analysis of acute exercise effects on state anxiety: an update of randomized controlled trials over the past 25 years. Depress Anxiety. 2015;32(8):624–34. 25899389 10.1002/da.22370

[35] Arapovic-Johansson B, Wåhlin C, Kwak L, Björklund C, Jensen I. Work-related stress assessed by a text message single-item stress question. Occup Med (Lond). 2017;67(8):601–8. 29016877 10.1093/occmed/kqx111 PMC5927000

[36] Garber CE, Blissmer B, Deschenes MR, Franklin BA, Lamonte MJ, Lee IM, et al. American College of Sports Medicine position stand. Quantity and quality of exercise for developing and maintaining cardiorespiratory, musculoskeletal, and neuromotor fitness in apparently healthy adults: guidance for prescribing exercise. Med Sci Sports Exerc. 2011;43(7):1334–59. 21694556 10.1249/MSS.0b013e318213fefb

[37] Olejnik S, Algina J. Generalized eta and omega squared statistics: measures of effect size for some common research designs. Psychol Methods. 2003;8(4):434–47. 14664681 10.1037/1082-989X.8.4.434

[38] Marcora SM, Staiano W, Manning V. Mental fatigue impairs physical performance in humans. J Appl Physiol (1985). 2009;106(3):857–64. 19131473 10.1152/japplphysiol.91324.2008

[39] Chaudhuri A, Behan PO. Fatigue and basal ganglia. J Neurol Sci. 2000;179(S 1–2):34–42. 11054483 10.1016/s0022-510x(00)00411-1

[40] Davis JM, Bailey SP. Possible mechanisms of central nervous system fatigue during exercise. Med Sci Sports Exerc. 1997;29(1):45–57. 9000155 10.1097/00005768-199701000-00008

[41] Olsson EM, Roth WT, Melin L. Psychophysiological characteristics of women suffering from stress-related fatigue. Stress Health. 2010;26(2):113–26.

[42] Ekkekakis P, Hall EE, Petruzzello SJ. Practical markers of the transition from aerobic to anaerobic metabolism during exercise: rationale and a case for affect-based exercise prescription. Prev Med. 2004;38(2):149–59. 14715206 10.1016/j.ypmed.2003.09.038

[43] Dishman RK, Thom NJ, Puetz TW, O’Connor PJ, Clementz BA. Effects of cycling exercise on vigor, fatigue, and electroencephalographic activity among young adults who report persistent fatigue. Psychophysiology. 2010;47(6):1066–74. 20409016 10.1111/j.1469-8986.2010.01014.x

[44] Crush EA, Frith E, Loprinzi PD. Experimental effects of acute exercise duration and exercise recovery on mood state. J Affect Disord. 2018;15(229):282–7. 10.1016/j.jad.2017.12.092 29329061

[45] Hallgren M, Vancampfort D, Hoang MT, Andersson V, Ekblom Ö, Andreasson S, et al. Effects of acute exercise on craving, mood and anxiety in non-treatment seeking adults with alcohol use disorder: An exploratory study. Drug Alcohol Depend. 2021;1(220):108506. 10.1016/j.drugalcdep.2021.108506 33461151

[46] Perkins SL, Cook DB, Herring MP, Meyer JD. Dose-response effects of acute exercise intensity on state anxiety among women with depression. Front Psychiatry. 2023;12(14):1090077. 10.3389/fpsyt.2023.1090077 PMC10213268 37252133

[47] Morava A, Dillon K, Sui W, Alushaj E, Prapavessis H. The effects of acute exercise on stress reactivity assessed via a multidimensional approach: a systematic review. J Behav Med. 2024;47(4):545–65. 38468106 10.1007/s10865-024-00470-w

[48] Hoffman MD, Hoffman DR. Exercisers achieve greater acute exercise-induced mood enhancement than nonexercisers. Arch Phys Med Rehabil. 2008;89(2):358–63. 18226663 10.1016/j.apmr.2007.09.026

[49] Bixby WR, Lochbaum MR. Affect responses to acute bouts of aerobic exercise in fit and unfit participants: An examination of opponent-process theory. J Sport Behav. 2006;29(2):111–25.

[50] Malmberg Gavelin H, Eskilsson T, Boraxbekk CJ, Josefsson M, Stigsdotter Neely A, Slunga JL. Rehabilitation for improved cognition in patients with stress-related exhaustion disorder: RECO - a randomized clinical trial. Stress. 2018;21(4):279–91. 29693483 10.1080/10253890.2018.1461833

[51] Reed J, Ones DS. The effect of acute aerobic exercise on positive activated affect: A meta-analysis. Psychol Sport Exerc. 2006;7(5):477–514.

[52] Geurts SA, Sonnentag S. Recovery as an explanatory mechanism in the relation between acute stress reactions and chronic health impairment. Scand J Work Environ Health. 2006;32(6):482–92. 17173204 10.5271/sjweh.1053

[53] AndersdotterSandström A, Fjellman-Wiklund A, Sandlund M, Eskilsson T. Patients with stress-induced exhaustion disorder and their experiences of physical activity prescription in a group context. Glob Health Action. 2023;16(1):2212950. 37314383 10.1080/16549716.2023.2212950 PMC10269406

[54] Harris S, Stratford P, Bray SR. Is it really worth the effort? Examining the effects of mental fatigue on physical activity effort discounting. J Sport Exerc Psychol. 2022;44(6):409–19. 36270628 10.1123/jsep.2021-0330

[55] Kwan BM, Bryan A. In-task and post-task affective response to exercise: translating exercise intentions into behaviour. Br J Health Psychol. 2010;15(Pt 1):115–31. 19397847 10.1348/135910709X433267 PMC12240157

[56] McDowell CP, Campbell MJ, Herring MP. Sex-related differences in mood responses to acute aerobic exercise. Med Sci Sports Exerc. 2016;48(9):1798–802. 27128666 10.1249/MSS.0000000000000969

[57] Hutchinson JC. Perceived effort and exertion. In: Z. Zenko & L. Jones Eds. Essentials of exercise and sport psychology: An open access textbook. Society for Transparency, Openness, and Replication in Kinesiology. 2021;294–315. 10.51224/B1013

