# Supplementary material for: Psychological responses to acute exercise in patients with stress-induced exhaustion disorder: a cross-over randomized trial
Source: BMC Psychiatry. 2025 Jan 24;25:72. doi: 10.1186/s12888-025-06484-1 (PMC11760732; doi:10.1186/s12888-025-06484-1)
Supplement: Supplementary file 1 — Additional file 1 [file 12888_2025_6484_MOESM1_ESM.pdf]

ADDITIONAL FILE 1: SUPPLEMENTARY MATERIAL

PSYCHOLOGICAL RESPONSES TO ACUTE EXERCISE IN PATIENTS WITH STRESS-INDUCED EXHAUSTION DISORDER - A CROSS-OVER RANDOMIZED TRIAL

Jenny Kling[1*], Robert Persson Asplund[1,3], Örjan Ekblom[1,4], and Victoria Blom[1,2]

[1] Department of Physical Activity and Health, The Swedish School of Sport and Health Sciences, Stockholm, Sweden. [2] Department of Clinical Neuroscience, Karolinska Institute, Stockholm Sweden.

[3] Department of Behavioural Sciences and Learning, Linköping University, Linköping, Sweden

[4] Department of Neurobiology, Care Sciences and Society, Division of Nursing, Research group: Health promotion among children and youth, Karolinska Institute, Stockholm, Sweden.

* Corresponding author: Jenny Kling, Department of Physical Activity and Health, The Swedish School of Sport and Health Sciences, Lidingövägen 1, SE-114 33 Stockholm, Sweden. E-mail: jenny.kling@gih.se

**Contents**	**Pages**
Table S1. Diagnostic Criteria for exhaustion disorder (ED) (F43.8 A) published by the National Board of Health and Welfare in Sweden	3
Table S2. Post hoc analysis of time x intensity interaction in perceived exertion	4
Table S3. Post hoc analysis of time x intensity x group interaction in fatigue	5
Table S4. Post hoc analysis of time x intensity x group interaction in energy	7
Table S5. Post hoc analysis of time effect in anxiety	9
Table S6. Post hoc analysis of time x group interaction in stress	10
Table S7. Means and Standard Deviations for Perceived Exertion (RPE) and Perceived Psychological Discomfort (SUDS) across Measurements and by Group and Intensity	11
Table S8. Means and Standard Deviations for Fatigue, Energy, Anxiety, and Stress across Measurements and by Group and Intensity	11
List of abbreviations	12

Table S1. *Diagnostic Criteria for exhaustion disorder (ED) (F43.8 A) published by the National Board of Health and Welfare in Sweden*[a]

A. Physical and mental symptoms of exhaustion during at least 2 weeks. The symptoms have developed in response to one or more identifiable stressors, which have been present for at least 6 months.

B. Markedly reduced mental energy, manifested by reduced initiative, lack of endurance, or increased time needed for recovery after mental efforts.

C. At least four of the following symptoms have been present most of the day, nearly every day, during the same 2-week period:

1. Persistent complaints of impaired memory and concentration.
2. Markedly reduced capacity to tolerate demands or to perform under time pressure.
3. Emotional instability or irritability.
4. Insomnia or hypersomnia.
5. Persistent complaints of physical fatigue and lack of endurance.
6. Physical symptoms such as muscular pain, chest pain, palpitations, gastrointestinal problems, vertigo, or increased sensitivity to sounds.

D. The symptoms cause clinically significant distress or impairment in social, occupational, or other important areas of functioning.

E. The symptoms are not due to the direct physiological effects of a substance (e.g., abuse of a drug or medication) or a general medical condition (e.g., hypothyroidism, diabetes, infectious disease).

---

[a] *All criteria with capital letters must be met to set the diagnosis.*

Table S2. *Post hoc comparisons of Time ✻ Intensity on perceived exertion (RPE)*

Comparison								
Time	Intensity	Time	Intensity	Mean Difference	SE	df	t	pbonferroni
Pre	Low	Pre	Mod	0.00123	0.0906	55.0	0.0136	1.000
		5min	Low	-5.64039	0.2582	55.0	-21.8482	**< .001**
		10min	Low	-6.50123	0.2648	55.0	-24.5535	**< .001**
		15min	Low	-6.84791	0.2832	55.0	-24.1809	**< .001**
	Mod	5min	Mod	-7.95197	0.1881	55.0	-42.2688	**< .001**
		10min	Mod	-8.60222	0.2034	55.0	-42.2937	**< .001**
		15min	Mod	-8.89963	0.2195	55.0	-40.5536	**< .001**
5min	Low	5min	Mod	-2.31034	0.1897	55.0	-12.1765	**< .001**
		10min	Low	-0.86084	0.1138	55.0	-7.5618	**< .001**
		15min	Low	-1.20751	0.1889	55.0	-6.3935	**< .001**
	Mod	10min	Mod	-0.65025	0.0991	55.0	-6.5647	**< .001**
		15min	Mod	-0.94766	0.1273	55.0	-7.4415	**< .001**
10min	Low	10min	Mod	-2.09975	0.2019	55.0	-10.4002	**< .001**
		15min	Low	-0.34667	0.1380	55.0	-2.5115	0.420
	Mod	15min	Mod	-0.29741	0.0942	55.0	-3.1577	0.072
15min	Low	15min	Mod	-2.05049	0.2005	55.0	-10.2266	**< .001**

Table S3. *Post hoc analysis of Time ✻ Intensity ✻ Group interaction on fatigue*

Comparison										
**Time**	Intensity	Group	Time	Intensity	Group	Mean Difference	SE	df	t	pbonferroni
Pre	Low	Control	Pre	Low	ED	-10.0810	1.297	56.0	-7.7715	**< .001**
			Pre	Mod	Control	0.7857	0.688	56.0	1.1426	1.000
			Post	Low	Control	0.8929	0.635	56.0	1.4054	1.000
			30min	Low	Control	1.2857	0.593	56.0	2.1678	1.000
			6h	Low	Control	0.1786	0.919	56.0	0.1944	1.000
			24h	Low	Control	0.2857	0.924	56.0	0.3093	1.000
		ED	Pre	Mod	ED	-0.1000	0.664	56.0	-0.1505	1.000
			Post	Low	ED	3.1667	0.614	56.0	5.1595	**< .001**
			30min	Low	ED	3.0667	0.573	56.0	5.3520	**< .001**
			6h	Low	ED	0.8667	0.888	56.0	0.9764	1.000
			24h	Low	ED	2.0000	0.892	56.0	2.2413	1.000
	Mod	Control	Pre	Mod	ED	-10.9667	1.198	56.0	-9.1523	**< .001**
			Post	Mod	Control	0.2500	0.749	56.0	0.3339	1.000
			30min	Mod	Control	-2.3929	0.682	56.0	-3.5068	0.171
			6h	Mod	Control	-0.1071	0.729	56.0	-0.1469	1.000
			24h	Mod	Control	0.0357	0.834	56.0	0.0428	1.000
		ED	Post	Mod	ED	3.5000	0.723	56.0	4.8394	**0.002**
			30min	Mod	ED	3.7667	0.659	56.0	5.7140	**< .001**
			6h	Mod	ED	1.0333	0.704	56.0	1.4669	1.000
			24h	Mod	ED	2.5000	0.806	56.0	3.1031	0.570
Post	Low	Control	Post	Low	ED	-7.8071	1.199	56.0	-6.5127	**< .001**
			30min	Low	Control	0.3929	0.457	56.0	0.8598	1.000
			6h	Low	Control	-0.7143	0.982	56.0	-0.7274	1.000
			24h	Low	Control	-0.6071	1.015	56.0	-0.5979	1.000
		ED	30min	Low	ED	-0.1000	0.441	56.0	-0.2265	1.000
			6h	Low	ED	-2.3000	0.949	56.0	-2.4244	1.000
			24h	Low	ED	-1.1667	0.981	56.0	-1.1892	1.000
	Mod	Control	Post	Mod	ED	-7.7167	0.941	56.0	-8.2001	**< .001**
			30min	Mod	Control	-2.6429	0.577	56.0	-4.5792	**0.005**
			6h	Mod	Control	-0.3571	0.904	56.0	-0.3950	1.000
			24h	Mod	Control	-0.2143	0.914	56.0	-0.2346	1.000
		ED	30min	Mod	ED	0.2667	0.558	56.0	0.4783	1.000
			6h	Mod	ED	-2.4667	0.874	56.0	-2.8238	1.000
			24h	Mod	ED	-1.0000	0.883	56.0	-1.1330	1.000
30min	Low	Control	30min	Low	ED	-8.3000	1.176	56.0	-7.0558	**< .001**
			6h	Low	Control	-1.1071	0.931	56.0	-1.1890	1.000
			24h	Low	Control	-1.0000	1.006	56.0	-0.9944	1.000
		ED	6h	Low	ED	-2.2000	0.900	56.0	-2.4456	1.000
			24h	Low	ED	-1.0667	0.972	56.0	-1.0979	1.000
	Mod	Control	30min	Mod	ED	-4.8071	0.904	56.0	-5.3169	**< .001**

			6h	Mod	Control	2.2857	0.661	56.0	3.4567	0.200
			24h	Mod	Control	2.4286	0.640	56.0	3.7933	0.070
		ED	6h	Mod	ED	-2.7333	0.639	56.0	-4.2788	**0.014**
			24h	Mod	ED	-1.2667	0.619	56.0	-2.0479	1.000
6h	Low	Control	6h	Low	ED	-9.3929	1.319	56.0	-7.1233	**< .001**
			24h	Low	Control	0.1071	0.671	56.0	0.1597	1.000
		ED	24h	Low	ED	1.1333	0.648	56.0	1.7481	1.000
	Mod	Control	6h	Mod	ED	-9.8262	1.218	56.0	-8.0646	**< .001**
			24h	Mod	Control	0.1429	0.745	56.0	0.1917	1.000
		ED	24h	Mod	ED	1.4667	0.720	56.0	2.0371	1.000
24h	Low	Control	24h	Low	ED	-8.3667	1.180	56.0	-7.0914	**< .001**
	Mod	Control	24h	Mod	ED	-8.5024	1.198	56.0	-7.0974	**< .001**

Table S4. *Post hoc analysis of Time ✻ Intensity ✻ Group interaction on energy*

Comparison										
Time	Intensity	Group	Time	Intensity	Group	Mean Difference	SE	df	t	pbonferroni
Pre	Low	Control	Pre	Low	ED	13.5548	1.440	56.0	9.4124	**< .001**
			Pre	Mod	Control	-1.2500	0.710	56.0	-1.7604	1.000
			Post	Low	Control	-3.8214	0.960	56.0	-3.9820	**0.038**
			30min	Low	Control	-1.1429	0.665	56.0	-1.7187	1.000
			6h	Low	Control	0.1429	0.764	56.0	0.1871	1.000
			24h	Low	Control	-2.0357	0.919	56.0	-2.2143	1.000
		ED	Pre	Mod	ED	0.2333	0.686	56.0	0.3401	1.000
			Post	Low	ED	-4.0000	0.927	56.0	-4.3143	**0.012**
			30min	Low	ED	-0.9333	0.642	56.0	-1.4529	1.000
			6h	Low	ED	-0.6333	0.738	56.0	-0.8585	1.000
			24h	Low	ED	-0.7000	0.888	56.0	-0.7881	1.000
	Mod	Control	Pre	Mod	ED	15.0381	1.369	56.0	10.9818	**< .001**
			Post	Mod	Control	-3.6071	0.970	56.0	-3.7178	0.089
			30min	Mod	Control	2.5357	0.758	56.0	3.3437	0.281
			6h	Mod	Control	-0.1786	0.802	56.0	-0.2226	1.000
			24h	Mod	Control	-2.2143	0.867	56.0	-2.5540	1.000
		ED	Post	Mod	ED	-6.4333	0.937	56.0	-6.8634	**< .001**
			30min	Mod	ED	-1.8000	0.733	56.0	-2.4569	1.000
			6h	Mod	ED	0.3667	0.775	56.0	0.4731	1.000
			24h	Mod	ED	-0.9333	0.838	56.0	-1.1143	1.000
Post	Low	Control	Post	Low	ED	13.3762	1.499	56.0	8.9237	**< .001**
			Post	Mod	Control	-1.0357	0.819	56.0	-1.2646	1.000
			30min	Low	Control	2.6786	0.835	56.0	3.2068	0.422
			6h	Low	Control	3.9643	1.032	56.0	3.8414	0.060
			24h	Low	Control	1.7857	1.189	56.0	1.5024	1.000
		ED	Post	Mod	ED	-2.2000	0.791	56.0	-2.7805	1.000
			30min	Low	ED	3.0667	0.807	56.0	3.8003	0.068
			6h	Low	ED	3.3667	0.997	56.0	3.3768	0.254
			24h	Low	ED	3.3000	1.148	56.0	2.8740	1.000
	Mod	Control	Post	Mod	ED	12.2119	1.596	56.0	7.6521	**< .001**
			30min	Mod	Control	6.1429	0.840	56.0	7.3129	**< .001**
			6h	Mod	Control	3.4286	0.947	56.0	3.6212	0.120
			24h	Mod	Control	1.3929	1.019	56.0	1.3669	1.000
		ED	30min	Mod	ED	4.6333	0.812	56.0	5.7094	**< .001**
			6h	Mod	ED	6.8000	0.915	56.0	7.4340	**< .001**
			24h	Mod	ED	5.5000	0.984	56.0	5.5868	**< .001**

Table S4. *Post hoc analysis of Time ✲ Intensity ✲ Group interaction on energy (continued)*

30min	Low	Control	30min	Low	ED	13.7643	1.452	56.0	9.4799	**< .001**
			30min	Mod	Control	2.4286	0.628	56.0	3.8689	0.055
			6h	Low	Control	1.2857	0.833	56.0	1.5435	1.000
			24h	Low	Control	-0.8929	0.913	56.0	-0.9783	1.000
		ED	30min	Mod	ED	-0.6333	0.606	56.0	-1.0444	1.000
			6h	Low	ED	0.3000	0.805	56.0	0.3728	1.000
			24h	Low	ED	0.2333	0.882	56.0	0.2646	1.000
	Mod	Control	30min	Mod	ED	10.7024	1.314	56.0	8.1426	**< .001**
			6h	Mod	Control	-2.7143	0.728	56.0	-3.7265	0.086
			24h	Mod	Control	-4.7500	0.927	56.0	-5.1216	**< .001**
		ED	6h	Mod	ED	2.1667	0.704	56.0	3.0790	0.611
			24h	Mod	ED	0.8667	0.896	56.0	0.9673	1.000
6h	Low	Control	6h	Low	ED	12.7786	1.473	56.0	8.6725	**< .001**
			6h	Mod	Control	-1.5714	0.789	56.0	-1.9916	1.000
			24h	Low	Control	-2.1786	0.762	56.0	-2.8598	1.000
		ED	6h	Mod	ED	1.2333	0.762	56.0	1.6179	1.000
			24h	Low	ED	-0.0667	0.736	56.0	-0.0906	1.000
	Mod	Control	6h	Mod	ED	15.5833	1.505	56.0	10.3518	**< .001**
			24h	Mod	Control	-2.0357	0.828	56.0	-2.4583	1.000
		ED	24h	Mod	ED	-1.3000	0.800	56.0	-1.6250	1.000
24h	Low	Control	24h	Low	ED	14.8905	1.606	56.0	9.2706	**< .001**
			24h	Mod	Control	-1.4286	0.851	56.0	-1.6792	1.000
		ED	24h	Mod	ED	4.44e- 15	0.822	56.0	5.40e-15	1.000
	Mod	Control	24h	Mod	ED	16.3190	1.351	56.0	12.0777	**< .001**

Table S5. *Post hoc analysis of Time effect on anxiety*

Comparison						
time	time	Mean Difference	SE	df	t	pbonferroni
pre	post	0.07201	0.0202	56.0	3.565	**0.008**
	30min	0.11617	0.0153	56.0	7.597	**< .001**
	6h	-0.04278	0.0211	56.0	-2.027	0.475
	24h	-0.03750	0.0247	56.0	-1.518	1.000
post	30min	0.04416	0.0151	56.0	2.934	**0.048**
	6h	-0.11479	0.0193	56.0	-5.934	**< .001**
	24h	-0.10951	0.0258	56.0	-4.241	**< .001**
30min	6h	-0.15895	0.0187	56.0	-8.488	**< .001**
	24h	-0.15368	0.0246	56.0	-6.242	**< .001**
6h	24h	0.00527	0.0207	56.0	0.255	1.000

Table S6. *Post hoc analysis of Time ✻ Group interaction on stress*

Comparison								
Time	Group	Time	Group	Mean Difference	SE	df	t	pbonferroni
Pre	Control	Pre	ED	-0.90357	0.2025	56.0	-4.4629	**0.002**
		Post	Control	0.25000	0.1177	56.0	2.1243	1.000
		30min	Control	0.19643	0.1026	56.0	1.9142	1.000
		6h	Control	-0.30357	0.1356	56.0	-2.2382	1.000
		24h	Control	-0.19643	0.1370	56.0	-1.4342	1.000
	ED	Post	ED	0.63333	0.1137	56.0	5.5705	**< .001**
		30min	ED	0.71667	0.0991	56.0	7.2290	**< .001**
		6h	ED	0.05000	0.1310	56.0	0.3816	1.000
		24h	ED	-0.01667	0.1323	56.0	-0.1260	1.000
Post	Control	Post	ED	-0.52024	0.1490	56.0	-3.4910	**0.043**
		30min	Control	-0.05357	0.0678	56.0	-0.7899	1.000
		6h	Control	-0.55357	0.1293	56.0	-4.2816	**0.003**
		24h	Control	-0.44643	0.1294	56.0	-3.4507	**0.048**
	ED	30min	ED	0.08333	0.0655	56.0	1.2719	1.000
		6h	ED	-0.58333	0.1249	56.0	-4.6701	**< .001**
		24h	ED	-0.65000	0.1250	56.0	-5.2005	**< .001**
30min	Control	30min	ED	-0.38333	0.1519	56.0	-2.5234	0.652
		6h	Control	-0.50000	0.1328	56.0	-3.7641	**0.018**
		24h	Control	-0.39286	0.1276	56.0	-3.0779	0.145
	ED	6h	ED	-0.66667	0.1283	56.0	-5.1950	**< .001**
		24h	ED	-0.73333	0.1233	56.0	-5.9470	**< .001**
6h	Control	6h	ED	-0.55000	0.2079	56.0	-2.6457	0.475
		24h	Control	0.10714	0.1301	56.0	0.8235	1.000
	ED	24h	ED	-0.06667	0.1257	56.0	-0.5304	1.000
24h	Control	24h	ED	-0.72381	0.1716	56.0	-4.2169	**0.004**

Table S7. *Means and Standard Deviations for Perceived Exertion (RPE) and Perceived Psychological Discomfort (SUDS) across Measurements and by Group and Intensity*

**Outcome & Group**	**Low**				**Moderate**			
	**Pre-Ex**	**5min-Ex**	**10min-Ex**	**15min-Ex**	**Pre-Ex**	**5min-Ex**	**10min-Ex**	**15min-Ex**
**Exertion (RPE)**								
ED	6.53 (1.04)	12.7 (2.02)	13.6 (2.03)	13.7 (2.15)	6.50 (1.14)	14.7 (1.31)	15.5 (1.55)	15.6 (1.64)
Control	6.0 (0.0)	11.1 (1.59)	11.9 (1.62)	12.5 (1.79)	6.07 (0.37)	13.8 (1.36)	14.4 (1.43)	14.7 (1.44)
**Discomfort (SUDS)**								
ED	1.57 (1.52)	2.1 (1.79)	2.0 (1.76)	2.0 (1.88)	1.6 (1.87)	2.41 (1.96)	2.17 (1.91)	2.14 (1.92)
Control	0.37 (0.77)	0.57 (0.9)	0.57 (1.01)	0.57 (1.14)	0.37 (0.72)	0.67 (1.12)	0.70 (1.32)	0.57 (1.28)

Table S8. *Means and Standard Deviations for Fatigue, Energy, Anxiety, and Stress across Measurements and by Group and Intensity*

**Outcome & Group**	**Low**					**Moderate**				
	Pre-Ex	Post-Ex	30mPost-Ex	6hPost-Ex	24hPost-Ex	Pre-Ex	Post-Ex	30mPost-Ex	6hPost-Ex	24hPost-Ex
**Fatigue (POMS)**										
ED	12.4 (6.57)	9.2 (6.2)	9.3 (6.08)	11.5 (6.68)	10.4 (5.82)	12.5 (6.2)	8.97 (4.67)	8.7 (4.47)	11.4 (6.03)	9.97 (6.17)
Control	2.27 (1.96)	1.47 (1.36)	0.97 (1.35)	2.07 (2.02)	1.87 (2.32)	1.4 (1.35)	1.17 (1.74)	3.83 (1.74)	1.59 (2.32)	1.41 (1.48)
**Energy (POMS)**										
ED	7.27 (4.44)	11.3 (5.97)	8.2 (5.38)	7.9 (5.35)	7.97 (6.48)	7.03 (4.94)	13.5 (6.71)	8.83 (4.82)	6.67 (5.7)	7.97 (5.33)
Control	20.8 (6.19)	24.4 (5.32)	22.0 (5.47)	20.5 (5.7)	22.8 (5.5)	22.1 (5.31)	25.7 (5.13)	19.5 (5.02)	22.3 (5.66)	24.2 (4.88)
**Anxiety (STAI-T)**										
ED	40.9 (8.68)	37.9 (8.13)	35.4 (6.59)	41.0 (9.22)	41.1 (7.91)	42.3 (9.72)	36.8 (8.57)	35.7 (7.73)	42.8 (7.52)	43.0 (8.71)
Control	27.4 (5.23)	26.1 (4.93)	25.3 (5.11)	29.5 (5.65)	28.3 (6.05)	26.7 (5.46)	25.9 (4.35)	24.7 (5.18)	29.0 (7.43)	28.9 (5.39)
**Stress**										
ED	2.4 (1.07)	1.83 (0.83)	1.6 (0.72)	2.23 (1.04)	2.30 (0.84)	2.3 (0.95)	1.6 (0.81)	1.67 (0.71)	2.37 (0.93)	2.43 (0.82)
Control	1.43 (0.63)	1.27 (0.52)	1.27 (0.58)	1.77 (0.82)	1.67 (0.71)	1.47 (0.68)	1.13 (0.35)	1.2 (0.48)	1.72 (0.88)	1.59 (0.68)

List of abbreviations

AD	Adjustment disorder
CRF	Cardiorespiratory fitness
DSM	Diagnostic and Statistical manual of mental Disorders
EBBS	Exercise Benefits/Barriers Scale
ED	Exhaustion disorder
GAD	Generalized anxiety disorder
GIH	The Swedish School of Sport and Health Sciences
HRV	Heart rate variability
ICD	International classification of diseases
KEDS	Karolinska Exhaustion Disorder Scale
ME/CFS	Myalgic encephalomyelitis/chronic fatigue syndrome
PA	Physical activity
PHQ	Patient Health Questionnaire
POMS	Profile of Mood States
PSQI	Pittsburgh Sleep Quality Index
RM-ANOVA	Repeated measures analysis of variance
RPE	Rating of perceived exertion
RPM	Revolutions per minute
SGPALS	Saltin-Grimby Physical Activity Level Scale
SISQ	Single Item Stress Question
SMBQ/M	Shirom-Melamed Burnout Questionnaire/Measure
STAI	The State-Trait Anxiety Inventory
SUD	Subjective Units of Distress
VAS	Visual analog scales
VO2 max	Maximal aerobic capacity
