# Supplementary material for: Psychological responses to acute exercise in patients with stress-induced exhaustion disorder: a cross-over randomized trial
Source: BMC Psychiatry. 2025 Jan 24;25:72. doi: 10.1186/s12888-025-06484-1 (PMC11760732; doi:10.1186/s12888-025-06484-1)
Supplement: Supplementary file 2 — Additional file 2 [file 12888_2025_6484_MOESM2_ESM.pdf]

ADDITIONAL FILE 2: SUPPLEMENTARY MATERIAL

PSYCHOLOGICAL RESPONSES TO ACUTE EXERCISE IN PATIENTS WITH STRESS-INDUCED EXHAUSTION DISORDER:

A CROSS-OVER RANDOMIZED TRIAL

Jenny Kling[1*], Robert Persson Asplund[1,3], Örjan Ekblom[1,4], and Victoria Blom[1,2]

[1] Department of Physical Activity and Health, The Swedish School of Sport and Health Sciences, Stockholm, Sweden.

[2] Department of Clinical Neuroscience, Karolinska Institute, Stockholm Sweden.

[3] Department of Behavioural Sciences and Learning, Linköping University, Linköping, Sweden

[4] Department of Neurobiology, Care Sciences and Society, Division of Nursing, Research group: Health promotion among children and youth, Karolinska Institute, Stockholm, Sweden.

* Corresponding author: Jenny Kling, Department of Physical Activity and Health, The Swedish School of Sport and Health Sciences, Lidingövägen 1, SE-114 33 Stockholm, Sweden. E-mail: jenny.kling@gih.se

Study Background Questionnaire

---

1. Date of Birth
[Field for input]

---

2. Gender

- Female
- Male
- Other

---

3. Marital Status

- Single
- Living apart together
- Married or cohabiting

---

4. How many children under the age of 18 live in your home for at least half the time?
[Field for input]

---

5. What is your highest level of completed education?

- Primary school or equivalent
- Secondary school or equivalent
- Post-secondary education less than 3 years (e.g., college, vocational training)
- Post-secondary education 3 years or more

---

6. What is your main occupation today?

- Working full-time
    - Occupation: [Field for input]
- Working part-time
    - Occupation: [Field for input]
    - Work percentage: [Field for input]
- Homemaker (caring for home and children)
- Unemployed
- Student

7. Are you currently on sick leave (full-time or part-time)?

- Yes
- No

8. What is your current sick leave percentage?

- 25%
- 50%
- 75%
- 100%

9. How long have you been on sick leave for this current condition? [Field for input]

10. How physically active are you during your leisure time?
If your activity varies greatly, such as between summer and winter, try to take an average. This question refers to the past year.

- Sedentary leisure time: Almost completely inactive; for example, reading, watching TV, using a computer, or other sedentary activities.
- Some physical activity during at least 4 hours per week: For example, cycling or walking to work, gardening, fishing, bowling, etc.
- Regular moderate physical activity and exercise at least 2-3 hours per week: For example, heavy gardening, jogging, swimming, aerobics, tennis, badminton, etc.
- Regular vigorous training or competitive sports (high-intensity activity): For example, running, skiing, swimming, football, handball, etc., several times a week.

11. Below are some statements related to perceptions of physical activity. Indicate to what extent you agree or disagree with the statement.

Statement	Fully agree	Agree	Disagree	Strongly disagree
I enjoy physical activity.	○	○	○	○
Physical activity reduces feelings of stress and tension for me.	○	○	○	○
Physical activity improves my mental health.	○	○	○	○
Physical activity makes me tired.	○	○	○	○

Statement	Fully agree	Agree	Disagree	Strongly disagree
Performing physical activity makes me feel relaxed.	○	○	○	○
I become exhausted from physical activity.	○	○	○	○
I get an increased sense of well-being from physical activity.	○	○	○	○
Physical activity gives me a sense of satisfaction from accomplishing something.	○	○	○	○
Physical activity is strenuous for me.	○	○	○	○

12. Do you currently have any acute infections or symptoms of illness (e.g., flu or cold)?

- Yes
- No

Do you use any asthma medication during physical activity?

- Yes
- No

---

13. Do you have any of the following conditions or diseases, either with or without medication?

- Gastrointestinal issues
- Diabetes
- Asthma
- Chronic Obstructive Pulmonary Disease (COPD)
- Other lung disease (please specify): [Field for input]
- High blood pressure/high cholesterol
- Cardiovascular disease
- Hypothyroidism
- Acute pain conditions
- Chronic pain conditions
- Fibromyalgia
- Chronic Fatigue Syndrome
- Post-COVID symptoms
- Dementia
- Bipolar disorder, type 1 or type 2
- Psychotic disorder
- Post-Traumatic Stress Disorder (PTSD)
- Obsessive-Compulsive Disorder (OCD)
- Social phobia
- Anorexia

- Bulimia
- Alcohol abuse
- Drug or medication abuse
- ADHD
- Other: Describe the condition and whether you currently have it or have had it in the past: [Field for input]

---

14. Do you take regular medication?

- Yes
- No

---

40. Medication?
If this question is answered **Yes**, please specify:

- Name of the medication, dosage, and how often it is taken (e.g., Citalopram, 50mg daily; Trombyl, 75mg daily).

Asthma medication
If you have asthma as indicated in the previous question:

15. Do you use asthma medication during physical activity?

- Yes
- No

---

16. Do you use contraceptives in the form of daily oral pills?

- Yes
- No

---

17. The following questions concern your sleep habits during the past month. Answer each question based on how it has typically been for you during the past month, including both weekdays and weekends. Answer all questions.

DURING THE PAST MONTH...

- What time have you usually gone to bed?
  [Field for input]
- How many minutes has it usually taken you to fall asleep in the evening?
  [Field for input]

- What time have you usually gotten up in the morning?
  [Field for input]
- How many hours of sleep do you usually get per night?
  [Field for input]

---

18. During the last month, how often have you had difficulty sleeping due to the following reasons?
(Scale: Never | Less than 1 time/week | 1-2 times/week | 3 or more times/week)

- Unable to fall asleep within 30 minutes.
- Waking up during the night or early morning.
- Having to go to the bathroom.
- Unable to breathe comfortably.
- Coughing or snoring heavily.
- Feeling cold.
- Feeling hot.
- Having unpleasant dreams.
- Experiencing pain in your body.
- Other reasons (please describe): [Field for input]

---

19. DURING THE PAST MONTH...
(Scale: **Never** | **Less than 1 time/week** | **1-2 times/week** | **3 or more times/week**)

- How often have you used sleeping pills to help you sleep?
- How often have you had difficulty staying awake while driving, eating, or socializing?
- How often have you found it hard to keep your enthusiasm up to get things done?

---

20. DURING THE PAST MONTH...

How would you describe your overall sleep quality?

- Very good
- Fairly good
- Fairly bad
- Very bad

---

21. In the past two weeks, how often have you been bothered by any of the following problems?
(Scale: Not at all | Several days | More than half the days | Nearly every day)

- Little interest or pleasure in doing things.
- Feeling down, depressed, or hopeless.
- Trouble falling or staying asleep, or sleeping too much.
- Feeling tired or having little energy.
- Poor appetite or overeating.

- Feeling bad about yourself — or that you are a failure or have let yourself or your family down.
- Trouble concentrating, such as reading the newspaper or watching television.
- Moving or speaking so slowly that others could have noticed. Or the opposite — being so fidgety or restless that you have been moving around a lot more than usual.
- Thoughts that you would be better off dead or thoughts of hurting yourself in some way.

---

22. Below are some statements that describe how one might feel. Read each statement carefully and then circle the number that best describes how you usually feel. There are no "right" or "wrong" answers.
Do not spend too much time on each statement—respond in the way that best describes how you usually feel.

(Scale: **Almost never** | **Sometimes** | **Often** | **Almost always**)

- I feel pleasant.
- I get tired quickly.
- I feel like crying.
- I wish I could be as happy as others seem to be.
- I miss out on things because I cannot decide fast enough.
- I feel rested.
- I am "calm and collected."
- I feel like problems grow so large that I cannot handle them.
- I worry too much about things that don't really matter.
- I am happy.
- I tend to take things too seriously.
- I lack self-confidence.
- I feel secure.
- I try to avoid dealing with difficulties and crises.
- I feel down.
- I am content.
- Some insignificant thought goes through my head and bothers me.
- I take disappointments so hard that I cannot let them go.
- I am a stable person.
- I get tense and upset when I think about my current concerns.

---

23. Below are a number of states that one can experience from time to time. Describe the extent to which you have experienced these during the past month.

(Scale: **1 = Almost never** to **7 = Almost always**)

- I feel tired.
- I feel physically exhausted.
- I feel like I've had enough.
- My "batteries" are "drained."
- I feel burned out.
- I feel like I don't have the energy to go to work in the morning.
- I have difficulty concentrating.
- I feel slow-witted and unable to think clearly.

- It feels difficult to think about complex matters.
- I feel scattered in my thoughts.
- I have difficulty remembering things.

---

24. The purpose of this form is to provide an overview of your current state.

We would like you to evaluate how you have felt over the past two weeks. The form contains a range of statements expressing varying degrees of discomfort, from no discomfort to extreme discomfort. Mark the box that best corresponds to how you have felt over the past two weeks.

Concentration
Please assess your ability to stay focused and concentrate. Think about how you perform in different tasks requiring varying levels of concentration, such as reading complex texts, reading light newspapers, or watching TV.

Scale:

- **0**: I do not have difficulty concentrating and can read, watch TV, and hold conversations as usual.
- **1**: Slight difficulty.
- **2**: Occasionally, I have trouble keeping my thoughts focused.
- **3**: Moderate difficulty.
- **4**: Often, I find it hard to concentrate.
- **5**: Severe difficulty.
- **6**: I am unable to concentrate on anything at all.

---

25. Memory

Here, we ask you to describe your ability to remember things. Think about whether you have difficulty remembering names, dates, or daily tasks.

Scale:

- **0**: I remember names, dates, and tasks I need to do.
- **1**: Occasionally, I forget unimportant things, but if I concentrate, I can usually remember.
- **2**: It happens that I forget things that are not very important, but I can recall them most of the time.
- **3**: Moderate difficulty; I often forget appointments or names of people I know well.
- **4**: I often forget significant tasks or people I know very well.
- **5**: I frequently forget important things or things I was supposed to do.
- **6**: I forget names, important dates, and daily tasks almost every day.

---

26. Physical Fatigability

This question concerns your physical stamina. Do you feel more physically tired than usual after daily activities or any form of physical exertion?

Scale:

- **0**: I feel as usual and perform physical activities in daily life or exercise as I normally do.
- **1**: Slightly more tired than usual but still manage my activities without major issues.
- **2**: Physical exertion feels more tiring than normal, but I can still perform my usual tasks.
- **3**: Moderate difficulty; I can manage daily activities but need to reduce intensity.
- **4**: I have trouble handling physical exertion. I can function as long as I move at a normal pace but struggle with increased intensity without feeling shaky and out of breath.
- **5**: Severe difficulty; I feel very weak and cannot even perform light physical activities or short distances.
- **6**: I feel extremely weak and unable to perform any physical activities.

---

27. Endurance

Here, we ask you to assess your endurance and whether you feel more mentally tired than usual in everyday situations.

Scale:

- **0**: I have as much energy as usual. I have no particular difficulty completing my daily tasks.
- **1**: Slightly more tired than usual but can still manage my tasks with minimal effort.
- **2**: I can carry out my daily tasks, but it requires more energy, and I get tired faster than usual. I need to take breaks more often than usual.
- **3**: Moderate difficulty; I get tired more often and need longer breaks to recover.
- **4**: I get unusually tired from trying to perform daily tasks, and social interactions with others exhaust me.
- **5**: Severe difficulty; I am too tired to do anything.
- **6**: I cannot perform any tasks.

---

28. Recovery

Here, we ask you to describe how well and how quickly you recover both mentally and physically after becoming fatigued.

Scale:

- **0**: I do not need to rest during the day.
- **1**: I feel tired during the day, but a short break is enough for me to recover.
- **2**: I feel tired during the day, but a small pause is sufficient to help me recharge.
- **3**: I feel moderately tired during the day and require longer breaks to recover.
- **4**: I feel very tired during the day and need long pauses to feel better.
- **5**: I feel extremely tired during the day and require frequent extended breaks.
- **6**: It does not matter how much I rest; it feels like I cannot recharge my batteries.

29. Sleep

Please describe how well you sleep. Think about the quality of your sleep and whether you feel rested during the past two weeks.

Scale:

- **0**: I sleep well and long enough to meet my needs and usually feel rested when I wake up.
- **1**: I sometimes sleep restlessly or wake up during the night and find it hard to go back to sleep. Occasionally, I do not feel rested after a night's sleep.
- **2**: I often sleep restlessly or wake up during the night and have difficulty going back to sleep. I frequently do not feel rested after a night's sleep.
- **3**: I sleep poorly almost every night, waking up often during the night and unable to fall back asleep. I rarely feel rested.
- **4**: I sleep poorly every night, waking up frequently and feeling unrested, regardless of how long I sleep.

30. Sensory Sensitivity

This question concerns whether you feel that one or more of your senses have become more sensitive to stimuli, such as sound, light, smells, or touch.

Scale:

- **0**: I do not feel that my senses are more sensitive than usual.
- **1**: Slightly more sensitive to sensory input than usual.
- **2**: Occasionally, sensory inputs such as light or sound bother me more than usual.
- **3**: Moderately sensitive to sensory inputs, with noticeable discomfort.
- **4**: Often sensitive to sensory inputs, such as loud sounds or bright lights.
- **5**: Highly sensitive to sensory inputs, significantly impacting my comfort.
- **6**: Extremely sensitive to sensory inputs, to the point that I must avoid them altogether.

31. Perceived Demands

Here, we ask you to reflect on how you respond to demands that you experience in everyday life. These demands can come from your surroundings or yourself.

Scale:

- **0**: I handle what I need or want to do without feeling it is particularly demanding or troublesome.
- **1**: Slightly more demanding than usual, but still manageable.
- **2**: Everyday situations that I previously managed without problems sometimes feel demanding and cause discomfort or stress.
- **3**: Moderately difficult; such situations now often feel demanding and cause significant discomfort or stress.

- **4**: Situations I previously handled without problems now feel demanding and often cause strong discomfort or stress.
- **5**: Most situations feel very demanding, and I find it difficult to handle them without feeling overwhelmed.
- **6**: Everything feels overwhelmingly demanding, and I cannot manage anything at all.

---

32. Irritation and Anger

This question concerns how irritable or angry you feel internally, regardless of whether you have shown it outwardly. Reflect particularly on how easily irritated you have been ("short-tempered") compared to what triggered it, and how often and intensely you have felt irritated or angry.

Scale:

- **0**: I do not feel irritated at all.
- **1**: I feel slightly irritable or angry, but it passes quickly.
- **2**: I feel more irritable or angry than usual, but it resolves on its own.
- **3**: Sometimes, I lose my temper in ways that are not normal for me.
- **4**: I get irritated or provoked more often than usual, and it lasts longer than it usually would.
- **5**: I often feel inwardly furious and have to make significant efforts to control myself.
- **6**: I feel constantly angry or irritable, and it severely impacts my ability to manage situations.

---

33. Do you currently smoke?

- Yes, daily
- Yes, occasionally
- No

---

34. Do you currently use snuff (smokeless tobacco)?

- Yes, daily
- Yes, occasionally
- No

---

35. How often do you consume alcohol?

- 4 or more times a week
- 2-3 times a week
- 2-4 times a month
- Once a month or less
- Never

---

36. On a typical day when you drink alcohol, how many "standard drinks" do you consume?
(1 standard drink = 4 cl of spirits, 8 cl of fortified wine, 12–15 cl of table wine, 33 cl of strong beer, or 50 cl of medium-strength beer)

- 10 or more
- 7-9
- 5-6
- 3-4
- 1-2

---

37. How often do you drink six or more "standard drinks" on one occasion?

- Daily or almost daily
- Weekly
- Monthly
- Less than once a month
- Never

---

38. Thank you for completing the questionnaire!
